# Injectable PTHF-based thermogelling polyurethane implants for long-term intraocular application

**DOI:** 10.1186/s40824-022-00316-z

**Published:** 2022-12-02

**Authors:** Kaiwen Zhang, Zengping Liu, Qianyu Lin, Yi Jian Boo, Valerie Ow, Xinxin Zhao, Daniel Soo Lin Wong, Jason Y. C. Lim, Kun Xue, Xinyi Su, Decheng Wu, Xian Jun Loh

**Affiliations:** 1 Institute of Materials Research and Engineering (IMRE), Agency for Science, Technology and Research (A*STAR), 2 Fusionopolis Way, Singapore, 138634 Singapore; 2 Department of Biomedical Engineering, Southern University of Science and Technology (SUSTech), 1088 Xueyuan Avenue, Shenzhen, 518055 People’s Republic of China; 3 Department of Ophthalmology, Yong Loo Lin School of Medicine, National University of Singapore, 1E Kent Ridge Road, Singapore, 119228 Singapore; 4 Institute of Molecular and Cell Biology (IMCB), Agency for Science, Technology and Research (A*STAR), 61 Biopolis Drive, Singapore, 138673 Singapore; 5 Singapore Eye Research Institute (SERI), 20 College Road, Singapore, 169856 Singapore; 6 Department of Ophthalmology, National University Hospital, 1E Kent Ridge Road, Singapore, 119228 Singapore; 7 Department of Materials Science and Engineering, National University of Singapore (NUS), 9 Engineering Drive 1, Singapore, 117576 Singapore

**Keywords:** Supramolecular hydrogel, Thermoresponsive, Vitreous substitutes, Implant, LCST polymer

## Abstract

**Background:**

Hydrogels show great potential to be used for intraocular applications due to their high-water content and similarity to the native vitreous. Injectable thermosensitive hydrogels through a small-bore needle can be used as a delivery system for drugs or a tamponading substitute to treat posterior eye diseases with clear clinical potential. However, none of the currently available thermosensitive hydrogels can provide intraocular support for up to 3 months or more.

**Method:**

In this study, an injectable polytetrahydrofuran (PTHF)-based thermosensitive hydrogel was synthesized by polyurethane reaction. We examined the injectability, rheological properties, microstructure, cytotoxicity, and in vivo compatibility and stability of the hydrogels in rabbit eyes.

**Results:**

We found that the PTHF block type and PTHF component ratio could modulate thermogelation properties of the polyurethane polymers. The PTHF-based hydrogel implants retained normal retinal structure and function. Incorporating bioinert PTHF generated highly biocompatible and more stable thermogels in the vitreous cavity, with gel networks and the presence of polymer still observed after 3 months when other thermogels would have been completely cleared. Moreover, despite lacking hydrolytically cleavable linkages, the polymers could be most naturally removed from the native vitreous by bio-erosion without additional surgical interventions.

**Conclusion:**

Our findings suggest the potential of incorporating hydrophobic bioinert blocks to enhance the in vivo stability of supramolecularly associated hydrogels for long-term intraocular applications.

**Graphical Abstract:**

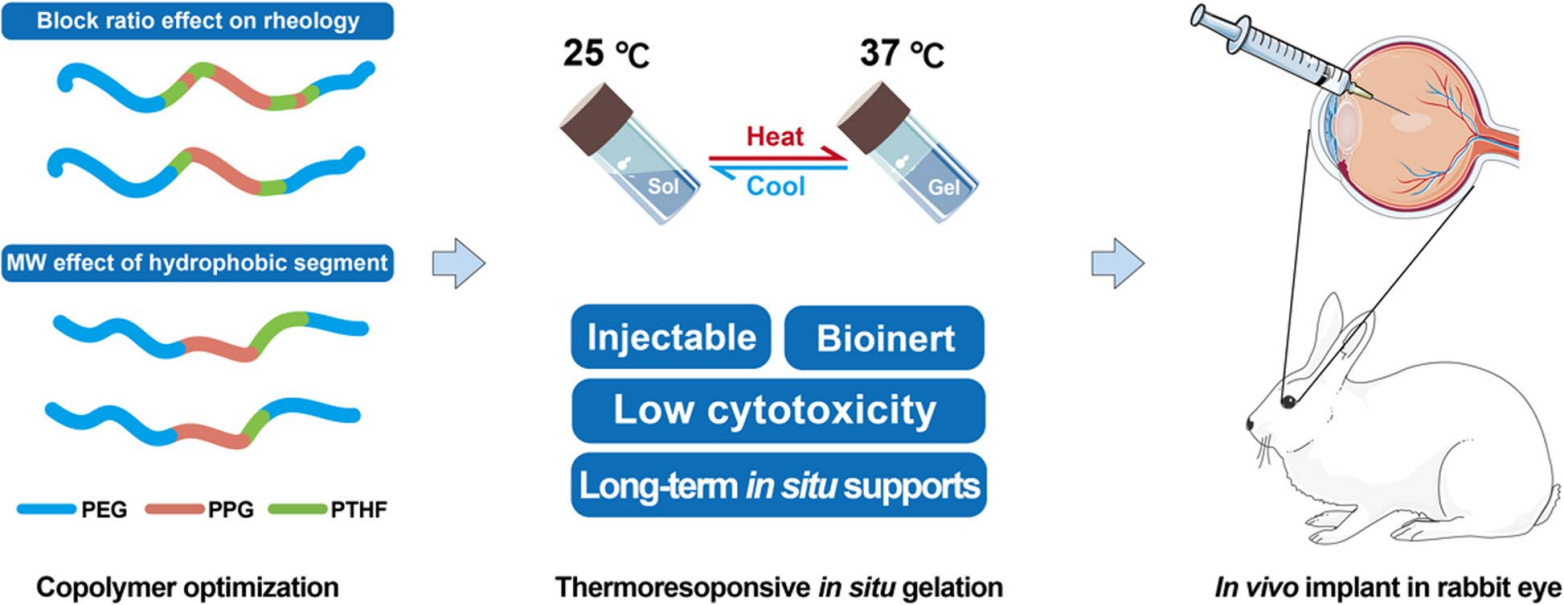

**Supplementary Information:**

The online version contains supplementary material available at 10.1186/s40824-022-00316-z.

## Introduction

The eye is a unique, complex, and highly important organ of the human body. According to the most recent report from World Health Organization (WHO), there are at least 2.2 billion people have a vision impairment, of whom at least 1 billion have a vision impairment that could have been prevented or is yet to be addressed globally [1]. Although less easily treatable, the three major blindness disease, age-related macular degeneration (AMD), diabetic retinopathy (DR) and glaucoma, collectively led to more than 19 in 86 million cases of moderate or worse vision impairment in adults aged 50 years and older in 2020 globally [2].

Vitrectomy is frequently performed on patients with DR or complicated retinal detachment and requires an artificial vitreous substitute (e.g. silicone oil) providing a long-term support up to a year [3, 4]. The silicone oil is the current gold standard clinical option, but with many shortcomings, such as retinal toxicity, absorption and potential development of cataracts, glaucoma and keratopathy, as well as the need for a secondary removal surgery [5, 6]. Therefore, newer materials are being investigated which can still provide support in the eye but with lower toxicity. The intravitreal injections of anti-vascular endothelial growth factors (anti-VEGF) has been established as the current standard clinical treatment for neovascular AMD. However, current treatment regime requires repeat monthly injection, which is a burden to the healthcare system [7]. Research and development efforts have increased using polymers-based materials for sustained drug release to the posterior region, showing promise results in drug delivery by increasing solubility, controlling pharmacokinetics and extending release [8]. Due to the high prevalence of eye disease but limited treatment options available, there is clinical unmet need in terms of developing novel biomaterials for intraocular therapeutic applications.

Hydrogels are a three-dimensional crosslinked polymeric matrix which can maintain its structure while preserving large amounts of water [9, 10]. With the capability to mimic the properties of extracellular matrices, cells, and soft tissues, hydrogels are widely used as biomedical implants and tissue substitutes [11, 12]. The vitreous body, filling the space between the lens and the retina, has a high water content (98%-99% water) and possesses prominent gelatinous structure [13, 14]. As such, hydrogel applications in ophthalmology are expected to be suitable as implant materials, such as vitreous substitutes and posterior transport implants. [15–17]. Thermogels, also known as inverse thermosensitive hydrogels, undergo a spontaneous sol-to-gel phase transition as the temperature increases [18–21]. Unlike chemically cross-linked hydrogels which may result in toxicity due to the cross-linker reactivity, in situ formed thermosensitive hydrogels are typically formed from amphiphilic block copolymers which physically crosslink to form into hydrogels [22–24]. By changing the ratio of hydrophilic block and hydrophobic blocks, the phase transition between room temperature and body temperature can be achieved in block copolymer thermogels. These thermogels have been widely used in drug delivery [25–28], 3D cell culture [29], tissue engineering [30], and deep tissue imaging [31]. Thermogels have unique advantages for use as an ocular implant: (1) quick in situ gelation property response to temperature change (2) high water content mimicking natural eye tissue (3) benign gelation behavior without chemical trigger, (4) easy to inject via a small-bore needle, and (5) potential to encapsulate drugs or small molecules as a drug delivery system. However, commercially available thermogel Pluronic-F127 have limitations including rapid erosion rate, easy collapse in aqueous environment, high gelation concentration, and retinal toxicity [32, 33]. The shortages of Pluronic-F127 are attributed to its low molecular weight and insufficient hydrophobicity, thus preventing the formation of strong micelle aggregation.

By introducing blocks with higher hydrophobicity, such as poly(ε-caprolactone) (PCL) [34], poly[(R)-3-hydroxybutyrate] (PHB) [35], and poly lactic acid (PLA) [36, 37], thermogels could be endowed with stronger physical crosslinks and adjustable mechanical properties. Although these units are easily biodegradable, for local implantation where long-term stability is required, some of them release acidic byproducts which potentially lead to an inflammatory response [38]. Polytetrahydrofuran (PTHF) is a synthetic polymer that has been reported to be used as a material for long-term biomedical applications with good biocompatibility [39, 40]. Compared with other hydrophobic materials that were reported, PTHF is a polyether that has a similar structure to poly(ethylene glycol) (PEG) and poly(propylene glycol) (PPG), and exhibits good chemical resistance, flexibility, hydrolytic stability and metabolic removability [41, 42]. PTHF has also been used as a modulating component of drug-loaded thermogel, which can control the lower critical solution temperature (LCST) of thermogel copolymer and significantly reduce the critical gel concentration (CGC) [43]. Our group has previously reported a degradable PCL-based thermogel as vitreous substitute for mid-term use (1 to 3 months) [44]. In addition to tamponade effects, we also showed the thermogels have intrinsic antiangiogenic properties, which may result in synergistic effects to be as a vitreous tamponade in DR patients [45]. In this study, we aim to develop a thermogel which could achieve long-term supports (over 3 months) in the vitreous and monitor the changes in the hydrogels after in vivo ocular implantation. Herein, we synthesized and characterized a series of multiblock supramolecular thermogels based on PEG/PPG/PTHF (termed as EPT) copolymers and identified a suitable block ratio with the optimal molecular weight range for ocular use. In vitro and in vivo tests have shown the optimal copolymer is of low cytotoxicity and could retain retinal function well for 3 months after implantation. This study suggests injectable PTHF-based thermogel is a promising implant for intraocular applications.

## Material and method

### Materials

Poly(ethylene glycol) (PEG) with *M*_n_ of 2050 g mol^−1^, poly(propylene glycol) (PPG) with *M*_n_ of 2000 g mol^−1^, poly(tetrahydrofuran) or poly(tetrahydrofuran carbonate) diol (PTHF) with *M*_n_ of 1000 and 2000 g mol^−1^, dibutyltin dilaurate (DBT) (95%), 1,6-hexamethylene diisocyanate (HMDI) (98%), 1,6-diphenyl-1,3,5-hexatriene (DPH), toluene(anhydrous), diethyl ether were purchased from Sigma-Aldrich (Singapore). All materials were used as received.

### Synthesis of PEG/PPG/PTHF copolymer

7.5 g PEG (*M*_n_ = 2050), 2.5 g PPG (*M*_n_ = 2000), 0.5 g PTHF (*M*_n_ = 1000) or PTHF carbonate (*M*_n_ = 2000) were weighed and added into a 250 mL round bottom flask, 30 mL anhydrous toluene was added and dissolved under magnetic stirring at 300 rpm for 20 min at 60 ℃ oil bath. Water in the sample was removed with toluene by azeotropic distillation using a rotary evaporator (BUCHI Rotavapor R-215) at a 55 ℃ water bath, 40 mbar air pressure. Stop evaporator when the toluene is fully evaporated. Repeated the process twice.

The samples were further dried under vacuum at 110 °C for 30 min, after which 10 µL DBT, 60 mL anhydrous toluene, and 0.86 mL HMDI were added sequentially. The samples were stirred at 300 rpm in an oil bath at 110 °C under dry argon atmosphere, reacted for 1 h. Then the reaction was terminated by adding 10 mL ethanol. The sample was stirred continuously and allowed to cool naturally to room temperature. The sample was poured into 1600 mL of ether to precipitate, then was filtered via suction filtration. Finally, dry nitrogen was used to blow the sample to obtain a powder-like white solid.

The solid powder was dissolved in isopropanol and poured into a dialysis tube (MWCO: 3.5 kDa) and dialyzed in large amounts of deionized water for 72 h. The dialyzed liquid was frozen in a -20 °C refrigerator and then lyophilized using a freeze dryer. The EPT copolymer product was obtained.

### Molecular characterization

1. Gel permeation chromatography (GPC): The test was performed to determine molecular weight using a gel permeation chromatography machine (Agilent 1260 Infinity II). 5 mg of EPT copolymer product was dissolved into 1 mL of THF (HPLC grade) and transfer the liquid to the GPC test vial using a 1 mL syringe while filtering using a PTFE (0.2 µm) filter tip. For the test, the same THF (HPLC grade) was used as the GPC eluent, and a monodisperse polystyrene standard sample was used to plot the correction curve. The experiment was run at a flow rate of 1.0 mL min^−1^.
2. Nuclear magnetic resonance hydrogen spectroscopy (^1^H NMR): The ^1^H NMR spectra of the EPT copolymer products were obtained by dissolving 10 mg of completely dried EPT product into 0.8 mL of deuterated chloroform, and the clarified liquid after complete dissolution was injected into a 5 mm NMR test tube for testing using a 500 MHz NMR instrument (JEOL 500 MHz NMR spectrometer) with single pulse test method at room temperature.

### Sol–gel transition determination

The sol–gel transition of EPT copolymers was determined using the test tube inversion method. Aqueous solutions of EPT copolymers at concentration range from 4.0 to 15.0 wt% were prepared in 4 mL glass vials and left to dissolve in a refrigerator at 4 °C for 24 h. The experimental temperature ranges from 0 to 74 °C with 2 °C intervals using an isopropanol bath and a water bath, each copolymer solution immersed for 5 min and inverted for 30 s. Gelation was defined as the formation of an intact and non-flowing gel after inversion. Phase diagrams were plotted using the recorded data to obtain the critical gel concentration (CGC). Here, CGC was defined as the lowest copolymer concentration at which stable gel formation could be observed.

### Micelle characterization

1. Determine the critical micelle concentration (CMC): CMC value of EPT thermogel copolymers were determined via the dye solubilization method. Gradient dilutions of 1 wt% solutions of EPT copolymer were prepared to obtain 4 mL of aqueous solutions of EPT copolymer at concentrations of 1, 0.5, 0.25, 0.1, 0.05, 0.025, 0.01, 0.005, 0.0025, and 0.001 wt%, respectively. DPH was dissolved in methanol and prepared as a 0.6 mM solution. To the gradient diluted copolymer solution, 20 µL of DPH solution was added separately and left for 3 h at room temperature under ventilation away from light to allow sufficient volatilization of methanol. The absorbance spectra were recorded using a UV–VIS spectrophotometer (SHIMDZU UV-3150 UV–VIS-NIR Spectrophotometer) at 25 ℃ in the range of 320 to 420 nm for measurement. The difference in absorbance at 378 and 400 nm was plotted against the logarithm of the concentration. The CMC value of the copolymer was determined from the intersection of the linear fits of the monomer and micellar systems.
2. Dynamic scattering light test: the micelle size of EPT thermogel was measured on a Zetasizer Nano ZS (Malvern Instruments, Southborough, MA) with laser light wavelength of 633 nm at a 173° scattering angle. 0.5 wt% of EPT copolymers were dissolved in DI water and measure temperature was set at 25 ℃. The micelle diameters of the EPT copolymer were given by the instrument.
3. Transmission electron microscopy: 20 µL EPT copolymer solution with the concentration of 0.5 wt% was dropped on a carbon-coated copper grid and dried overnight. Then the TEM image was taken at an acceleration voltage of 100 keV (Hitachi HT7700).

### Rheology measurement

The EPT thermogels were formulated by dissolving the multiblock copolymers in deionized water and equilibrium at 4 ℃ overnight. The rheological properties were measured using a TA Instruments Discovery Hybrid Rheometer Series (DHR-3, Research Instruments, Inc.) installed with a 40 mm parallel plate geometry. Temperature of thermogel samples were controlled by a temperature-controlled Peltier plate system. The gap was set at 900 µm. In temperature sweep protocol, rheological properties include storage modulus (G′), loss modulus (G′′) and complex viscosity were determined from 5 to 40 ℃ with heating rate 3 ℃ min^−1^, strain set at 1% and frequency fixed at 1 Hz. In flow sweep protocol, the relation between shear rate, stress, and viscosity was investigated by changing shear rate from 0.01 to 125 1/s at 25 ℃.

### Field emission scanning electron microscopy

Aqueous solutions of EPT copolymer at concentrations of 5.0, 7.0, 9.0, and 11.0 wt% were prepared and placed in a refrigerator at 4 ℃ overnight to dissolve completely. The gel was formed by equilibration under a constant temperature water bath at 37 ℃ for 2 h. The gel was then flash frozen using liquid nitrogen to ensure that the microstructure of the gel at 37 ℃ was retained. Freeze-drying was performed for 3 d to completely remove water. The gel specimens were cross-sectioned and sputter-coated with gold using a plating machine (JEOL JFC-1200 FINE COATER) at 30 Pa for 40 s under vacuum to neutralize its charge effects. The microstructures of the gels were visualized using a field emission scanning electron microscope (JEOL JSM-6700F Field emission electron microscope) at 1000 × and 5000 × magnification.

### Hydrogel erosion study

For gel erosion study, hydrogels were prepared in pre-weighed glass vials. 70 mg or 100 mg of EPT copolymer was dissolved in 1 mL of DI water in a 4 mL glass vial at 4 ℃ overnight to form a solution at 7 wt% and 10 wt% respectively. The EPT solutions were incubated in a 37 ℃ oven for 2 h for gel formation. An additional 2 mL of pre-warmed water was added on top of the gels, and the whole set up was placed on an orbital shaker at 350 rpm in a 37 ℃ oven. At regular time points, the supernatant was removed from the sample glass vial and freeze dried. The mass of the gel was also measured by weighing the sample glass vials without its supernatant liquid. After mass measurements, 2 mL of DI water was replaced into the sample glass vials, and the set up was placed back onto the orbital shaker in the 37 ℃ oven. To determine the dry mass, the mass after freeze drying of the supernatant was used. To determine the wet mass, the mass of the pre-weighed empty vial was subtracted from the total mass of the wet gel in the vial. The measured wet masses and cumulative dry masses were then plotted against time respectively.

### *In vitro* release assay

Bovine serum albumin (BSA) was dissolved in phosphate buffer saline at 40 mg/mL. 10 mg of copolymer were placed in 1.5 mL centrifuge tubes and 100 µL of BSA solution was added. The solutions were mixed thoroughly and dissolved for 3 nights at 4 °C to form drug encapsulated hydrogels. The prepared BSA-hydrogels were transferred to 37 ℃ for half an hour for hydrogel formation, 1 mL of pre-warmed fresh PBS was added, and the tubes were kept at 37 ℃ with shaking at 50 rpm. At certain intervals, 500 µL of supernatants were removed followed by addition of an equal volume of pre-warmed fresh PBS. The collected supernatants were kept at -20 °C for protein quantification. Protein quantification was carried out using the Pierce microBCA Protein Assay kit (Thermo Fisher Scientific, Waltham, MA). Quantitation of protein amount was based on a calibration curve, obtained with the corresponding BSA stock solution, in the range of 20 – 2000 µg/mL.

### *In vitro* gel optical performances

Dissolve EPT copolymer with deionized water, the transparency of 7 wt% EPT copolymer in solution status and gel status was checked by the visibility of A*STAR logo placed behind, and the transmittance was further tested at 25, 37, and 60 ℃ using SHIMDZU UV-3150 UV–VIS-NIR Spectrophotometer. EPT thermogels were placed in water bath with set temperature for 10 min to equilibrium. The range of measurement set at 400 to 700 nm, using water as baseline and silicone oil as comparison.

Refractive index of the thermogels were measured using an Abbe refractometer (NAR-1 T SOLID, ATAGO, Japan). Each thermogel was pipetted onto the glass measurement platform and equilibrated at the desired temperature for 2 min. The color conversion knob was turned until a clear boundary line was seen, and the refractometer knob was adjusted until the boundary line was aligned at the crosshairs. The refractive index was then directly read off the machine.

### *In vitro* cytotoxicity test

The adult retinal pigment epithelial (ARPE-19) cell line was cultured in Dulbecco’s Modified Eagle’s medium (DMEM) supplemented with 10% fetal bovine serum (FBS) and 100 U mL^−1^ concentrations of penicillin and streptomycin. The culture vessel was a T-75 slant-mouth culture flask in a humidified incubator (Sanyo CO_2_ Incubator, Model MCO 18AC) containing 5% CO_2_ at 37 °C. The culture medium was changed daily until the cells covered 90% of the bottom of the culture flask.

The EPT copolymer was dissolved in PBS with 1, 5 and 7 wt% concentration and their pH were then measured using a pH meter, a portion of the samples were taken and stored in 4 ℃ and 37 ℃ for 8 weeks and measured their pH every 2 weeks (*n* = 3). For 7 wt% gel, add same volume of PBS at 37 ℃, soak 24 h to obtain leachate. The cultured cells were digested, centrifuged, and resuspended in DMEM. Seed cell in a 96-well plate at the density of 5,000 cells per well in 100 µL culture medium and incubated for 24 h. Afterwards, additional 100 µL of PBS, EPT-1 wt%, EPT-5 wt% and EPT-7 wt%’s leachate were added separately, then incubated for another 24 h. Discard original medium, then 100 µL fresh medium with 10% of CCK-8 reagent was added to wells and incubated for 2 h before reading the OD value at the 450 nm under a plate reader.

#### Animals

Six New Zealand white rabbits (body weight 2.8 – 3.2 kg) were obtained from BioSystems (BioSystems Corporation Pte Ltd., China). All experiment designs and procedures were reviewed and approved by the Institutional Animal Care and Use Committee of the SingHealth (Singapore). All animals were treated in accordance with the Association for Research in Vision and Ophthalmology (ARVO) Statement for the Use of Animals in Ophthalmic and Vision Research.

#### Intravitreal injection of EPT thermogel in rabbit eyes

Optimized EPT copolymers were dissolved at 7 wt% in balanced salt solution for ophthalmic irrigation (AMO® ENDOSOL, Abbott) in a tissue culture hood and stored in a prefilled 3 mL syringe with an air-tight cover. 20 wt% Pluronic F127 polymer solutions were also prepared as a control. The solutions were further treated under ultraviolet (ultraviolet power: 281 μW cm^−2^, at 254 nm wavelength) for 3 min before animal surgeries. The EPT thermogels were intravitreally injected in vitrectomized rabbit eyes [44]. Animals were sedated with ketamine (50 mg kg^−1^, body weight (BW)) and xylazine (10 mg kg^−1^, BW). Pupils were dilated with an application of 1% tropicamide and 2.5% phenylephrine. A 25-gauge (G), 3-ports trans pars plana vitrectomy was performed using a Stellaris Elite (Bausch & Lomb, Singapore) vitrectomy machine and a surgical microscope (OPMI-Lumera 700, C. Zeiss Meditec, Singapore). Triamcinolone was used to have a better vitreous visualization during posterior vitreous detachment introduction. Thereafter, 0.5 to 0.8 mL of 7 wt% EPT gels (*n* = 6) or 20 wt% Pluronic F127 (*n* = 3) were injected through a 25 G cannula into vitreous cavity. A normal intraocular pressure was confirmed at the end of the surgery before the sclerotomies were sutured with 7–0 vicryl sutures. Topical antibiotic and steroid ointment (tobramycin and dexamethasone) were applied to the treated eyes twice a day for 5 days post-surgery.

#### Post-operative follow up by ophthalmic multimodal assessments

The clarity and biocompatibility of the EPT thermogels in the rabbit vitreous cavity were monitored non-invasively through the clear optical media of the eye. The animals were sedated, and pupils were dilated prior to the assessments as described above. The cornea and color fundus images were taken from a biomicroscope (Righton) equipped on a slit-lamp. Real-time photography of the retina by a confocal scanning laser ophthalmoscope (infrared reflectance/IR fundus) and optical coherence tomography (OCT) images (cross section of retinal layers) were examined non-invasively using spectral domain Optical Coherence Tomography (SD-OCT, Heidelberg engineering, Heidelberg, Germany). Two independent graders measured the full retinal thickness based on OCT images using built in Heidelberg software (*n* = 10 measurements from each image, three representable images at each time point per rabbit) [46]. All animals had repetitive multimodal imaging at baseline, postoperative days 7, 14, 30, 60 and 90. Intraocular pressure was measured through the centre of the cornea by TonoPen XL tonometer (Reichert Ophthalmic Instruments) at baseline, days 7, 14, 30, 60 and 90.

Full field electroretinography (ERG) was used to assess retinal function at baseline and monthly post-surgery. Animals were kept in dark adapt for at least 20 min before recordings. The assessments were performed by an Espion system (Diagnosis LLC, USA) with protocols and procedures based upon those recommended for humans by the International Society for Clinical Electrophysiology of Vision (ISCEV) [47].

#### Harvesting of vitreous

Animals were sacrificed in deep anesthesia at 3-month post operation. The eye globes (*n* = 3, 7 wt% EPT gel) for polymer and gel analysis were directly enucleated. After removing the conjunctiva and muscles, the globes were washed throughout with PBS 5 times. Then a 19 G needle was used to perforate into the vitreous cavity to withdraw vitreous avoiding contamination with surrounding tissues. The harvested vitreous were kept at -20 ℃ before polymer and gel analysis.

#### Histology

The harvested eye globes for histology (*n* = 3, 7 wt% EPT gel; *n* = 3, 20 wt% Pluronic F127) were perfused and fixed with 10% formalin. After enucleation, and entire globes were further fixed in the same fixative overnight. Full-thickness samples (retina → sclera) at posterior poles (approximate 5 × 5 mm) were collected and embedded in paraffin. Sections were cut at 10 μm with a microtome (Leica RM2255; Leica, Wetzlar, Germany) and stained with hematoxylin and eosin (H&E).

#### Analysis of remnant EPT thermogel in harvested vitreous

The harvested vitreous was split into several portions for further gel and polymer analysis. For SEM analysis, 200 µL of vitreous was warmed up at 37 ℃ for 2 h, and flash frozen using liquid nitrogen to retain the microstructure, and SEM performed after freeze drying. For water suppression ^1^H NMR, 150 µL of vitreous was mixed with 600 µL of deuterated water, and water suppression conducted (δ = 4.68 ppm). For quantitative ^1^H NMR, 200 µL of vitreous was freeze dried, and then mixed with 1 mL of chloroform-d containing 5 mg mL^−1^ of benzoic acid. The benzoic acid of known concentration with its characteristic peak (δ = 8.11 ppm) acted as a reference and was integrated in comparison with the characteristic peaks of the EPT polymer (PEG, δ = 3.63 ppm and PPG, δ = 1.12 ppm) so that the concentration of EPT polymer present could be determined. The remnant gel percentage was determined by normalising the calculated EPT polymer concentration to the initial EPT concentration at implantation. For GPC, one portion of vitreous was mixed with 3 portions of chloroform. The organic fraction was isolated and dried down. The dried organic fraction of vitreous was mixed with 1 mL of THF solvent, filtered and run on a GPC.

#### Statistical analysis

Data on retinal thickness measurements based on OCT images are presented as the mean ± SD, statistical analysis was performed using paired *t* test in GraphPad software (V8.2.1, GraphPad software Inc.) with the level of significance set at 0.05.

## Results

### Synthesis and characterization of EPT copolymers

**Fig. 1:**
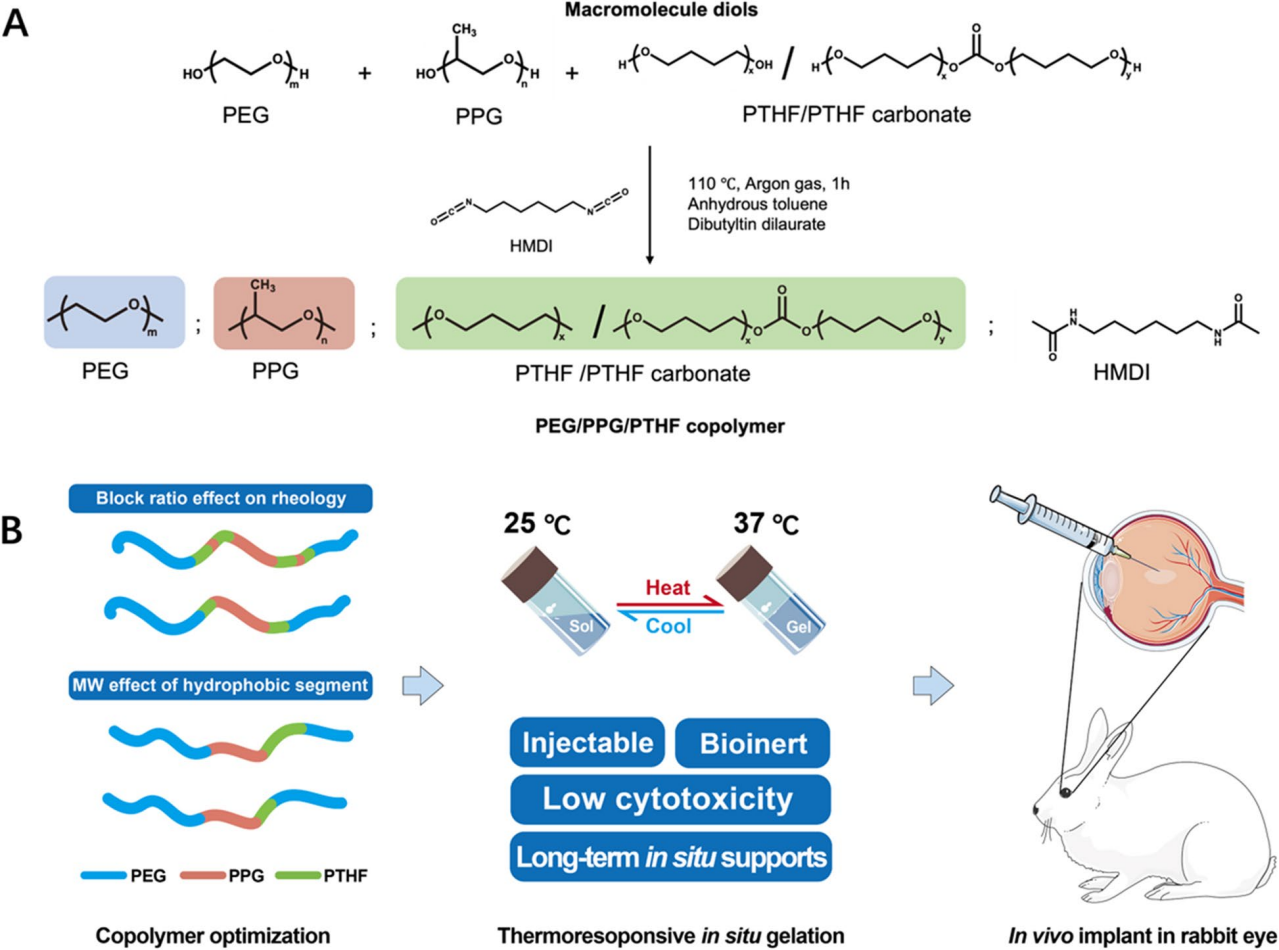
**A** Synthesis of thermogelling polyurethanes with PTHF. The degree of PTHF conjugation can be varied by changing feed ratios. **B** Schematic for thermogels based on bioinert PTHF for intraocular application. These hydrogels are sustainably sourced with low cytotoxicity and long-term in situ supports

We synthesized amphiphilic multi-block copolymers of poly (PEG/PPG/PTHF urethane, termed as EPT) with different macromolecular diol blocks from before [44, 48], and the overall scheme was shown in Fig. 1. In brief, the macromolecular diols were covalently linked using hexamethylene diisocyanate (HMDI) as the coupling reagent and dibutyltin dilaurate (DBT) as the catalyst. The copolymer products were composed of hydrophilic block (PEG), thermosensitive block (PPG), and hydrophobic block (PTHF).

By adjusting the feed ratio, we obtained a series of PTHF-based copolymers with different amounts and molecular weight of PTHF incorporated (Table 1). The PEG/PPG feed ratio was fixed at 3:1 by mass to ensure that the EPT copolymer contained sufficient hydrophobic PPG blocks for thermogelation, while PTHF feed ratios were varied. For this paper, we adopted the following nomenclature when naming the EPT polymers: the last number represents the PTHF content (in wt%), while 1 T and 2 T represents 1 kDa PTHF and 2 kDa PTHF-carbonate respectively. The ^1^H NMR spectra of the synthesized EPT samples indicate the successful incorporation of PTHF into the polyurethane copolymer (Supplementary Figure S1). By integrating the characteristic peaks of PEG (δ = 3.62), PPG (δ = 1.12) and PTHF (δ = 1.60), the actual weight percentage of EPT in the copolymer can be determined. In our previous work with PEG/PPG/PCL (EPC) thermogel, it was found that an overly high or low polymer concentration could lead to retinal toxicity, and an optimal molecular weight of 40 to 50 kDa showed the best performance in rabbit eye model [44, 49]. In this study, reaction time, temperature, and polymer concentration were controlled to ensure that molecular weights remain in the optimal range.

**Table 1:** Molecular characteristics of the EPT copolymers of different feed ratios

	**Feed ratio (g)**			**Composition(wt%)**^**a**^			**Molecular weight**^**b**^		
Sample	PEG	PPG	PTHF	PEG	PPG	PTHF	*M*_n_ (kDa)	*M*_w_ (kDa)	PDI
EPT3-1 T	7.5	2.5	0.3	75.77	21.31	2.92	40.8	54.8	1.34
EPT3-2 T	7.5	2.5	0.3	76.81	20.25	2.93	43.5	56.7	1.30
EPT5-1 T	7.5	2.5	0.5	73.62	20.71	5.67	46.6	65.8	1.41
EPT5-2 T	7.5	2.5	0.5	75.70	19.96	4.34	47.2	63.6	1.34
EPT7-1 T	7.5	2.5	0.7	74.49	18.33	7.18	46.4	61.9	1.33

^a^ Composition was determined by integration of the ^1^H NMR spectra for PEG, PPG and PTHF at their characteristic resonance peaks of δ = 3.60, 1.10, and 1.60 respectively

^b^ Molecular weight was determined using gel permeation chromatography with THF as solvent and polystyrene standards

### Micellisation and thermogelation properties

The amphiphilic EPT copolymers showed a tendency to form into micelles when dissolved in water. When the hydrophobic block PTHF was introduced in the polymer chain, we observed that the critical micellisation concentration of EPT copolymers were all reduced to below about 0.2 wt% (Table 2, Supplementary Figure S2). The CMC of EPT copolymers decreases with increasing copolymer hydrophobicity, which is consistent with previous reports [50]. The hydrodynamic radius of the micelles was further studied using dynamic light scattering. EPT-2 T copolymers possessed a larger micelle size than EPT-1 T, and the effect was especially obvious in EPT5, where EPT5-2 T showed a micelle size of 98.2 ± 0.16 nm which was twice as large as EPT5-1 T (45.5 ± 0.21 nm). The morphology and size of the EPT5-1 T micelles observed in the TEM images are consistent with the DLS results (Figure S3).

**Table 2:** CGC, CMC, and micelle size of EPT Copolymers

Sample	CGC (wt%)	CMC (wt%)	Micelle size (nm) ^a^
EPT3-1 T	11	0.20	40.2 ± 0.34
EPT3-2 T	9	0.14	49.1 ± 0.14
EPT5-1 T	5	0.15	45.5 ± 0.21
EPT5-2 T	5	0.11	98.2 ± 0.16
EPT7-1 T	6	0.11	43.5 ± 0.14

^a^ Determined by DLS at 0.5 wt% at 25 °C

**Fig. 2:**
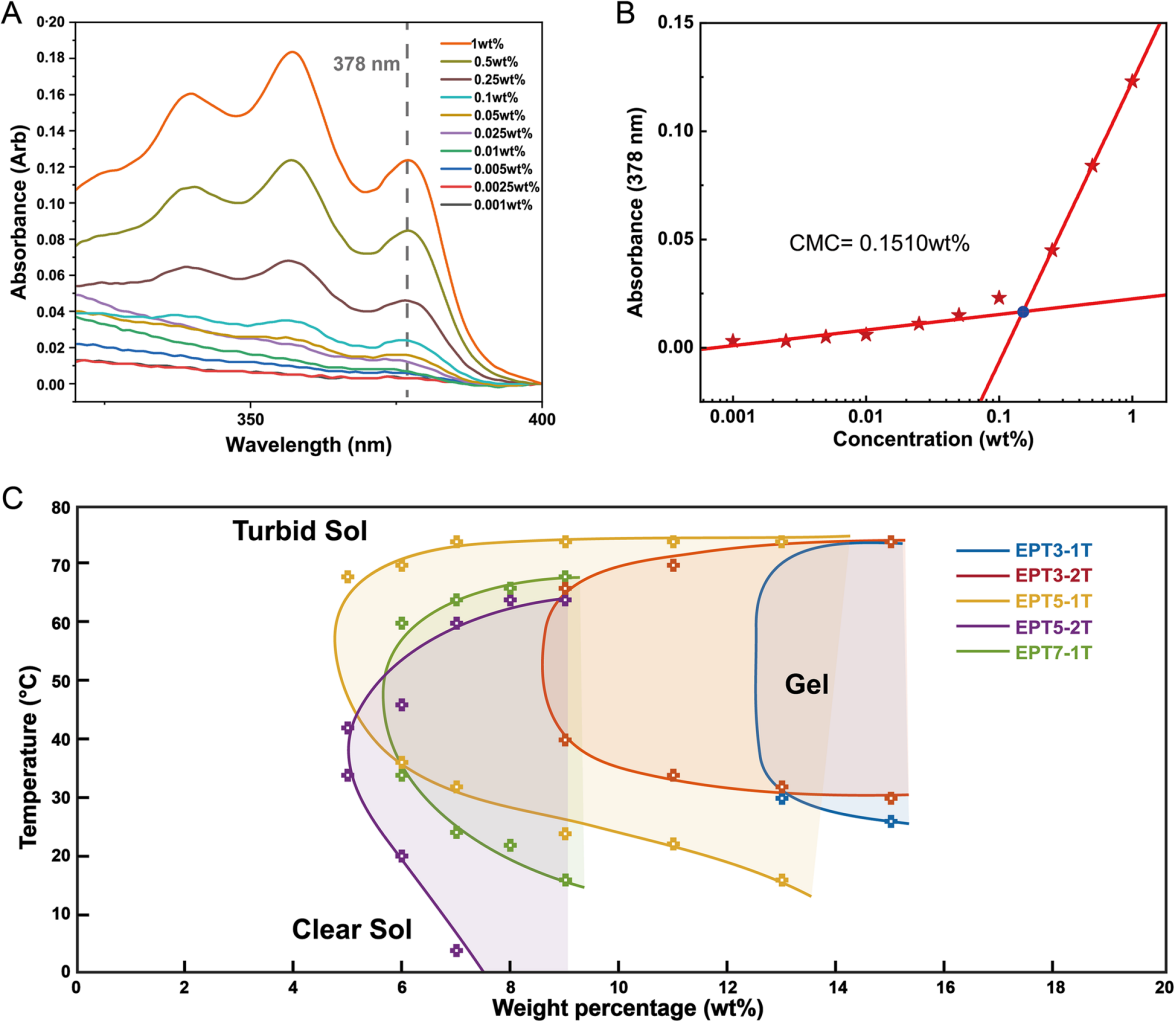
Micellisation and sol–gel transition phase diagram. **A** Representative UV–vis spectra of DPH with increase concentration of EPT5-1 T in water; **B** Determination of the CMC of EPT5-1 T based on absorbance at 378 nm. **C** Phase diagram of EPT copolymers

To investigate the gelation behavior of those EPT thermogels, the tube inversion test was employed to determine the CGC, the results were illustrated and compared in a phase diagram (Fig. 2). As the content of hydrophobic block PTHF increased, a significant decrease in CGC was observed. Using 2 kDa PTHF imparted a higher hydrophobicity to copolymer compared to 1 kDa PTHF, which led to a lower CGC for EPT3 and a lower gelation temperature for EPT5. This indicated that the hydrophobicity became more pronounced when the PTHF content increased due to the higher molecular weight of the hydrophobic block. However, the lowest value of CGC was limited at around 5 wt%. A stable gel could not be formed if the EPT copolymer concentration fell below 5 wt%.

**Fig. 3:**
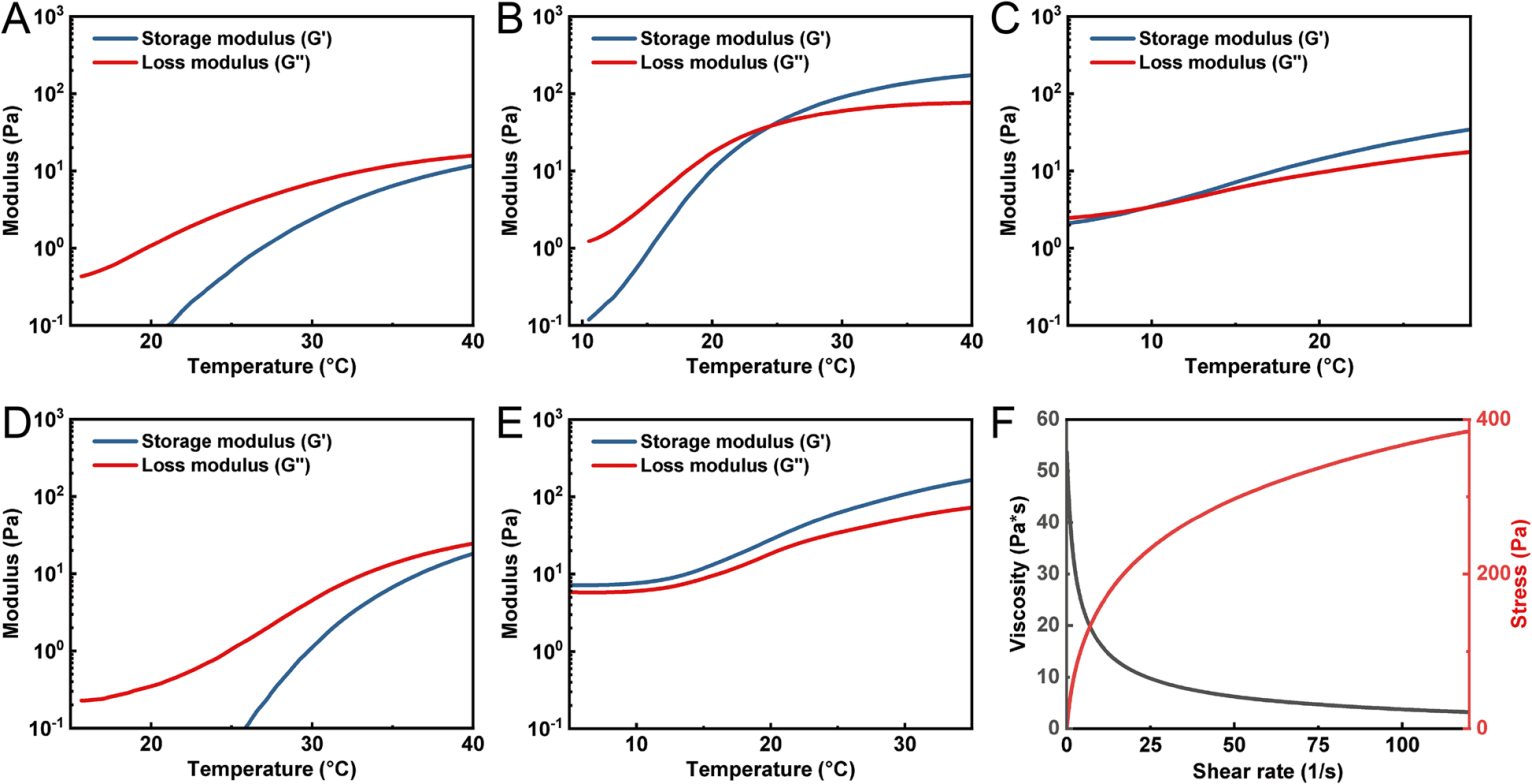
Rheological characterisation of EPT thermogels (7 wt%). Oscillatory temperature sweep of **A** EPT3-1 T; **B** EPT5-1 T; **C** EPT7-1 T; **D** EPT3-2 T; **E** EPT5-2 T. Flow sweep of (F) EPT5-1 T at 25 ℃

Excessively high copolymer concentrations could form firm gel with high swelling, leading to increased intraocular pressure and inflammation, while low copolymer concentrations may not be able to provide tamponade effect and lead to cytotoxicity due to the onset of surfactant effects [44]. Therefore, in this study, the copolymer concentration was fixed at 7 wt% and the rheological properties of EPT thermogels were characterized at this concentration (Fig. 3). The composition of PTHF has a crucial effect on the gelation temperature, storage modulus (G'), and complex viscosity of the gel. To match the mechanical properties of the vitreous humor, the optimal G' of hydrogel implant is around 100 to 200 Pa. This modulus range is similar to the native vitreous humor and can maintain shape during rapid eye movement [49]. At the optimal molecular weights, EPT copolymers with low or high PTHF ratio did not form thermogels with suitable mechanical properties at the target concentration. Neither 7 wt% EPT3-1 T nor 7 wt% EPT3-2 T convert to a gel at body temperature. 7 wt% EPT7-1 T formed a gel at 9.6 ℃, a temperature significantly lower than room temperature. Whereas 7 wt% EPT5-1 T showed a gelation temperature of 24.5 ℃ and storage modulus of around 150 Pa, while 7 wt% EPT5-2 T showed a storage modulus above loss modulus in the test temperature range, indicating that it is in a gel state at all the temperatures tested. EPT5-1 T also showed a shear thinning behavior at room temperature, so it could be injected via a small-bore needle. Considering the current characterization results, 7 wt% EPT5-1 T is the most suitable composition among the above PTHF-based thermogels for further implantation in rabbit eyes.

### Characterisation of 7 wt% EPT5-1 T thermogel for suitability in vitro

**Fig. 4:**
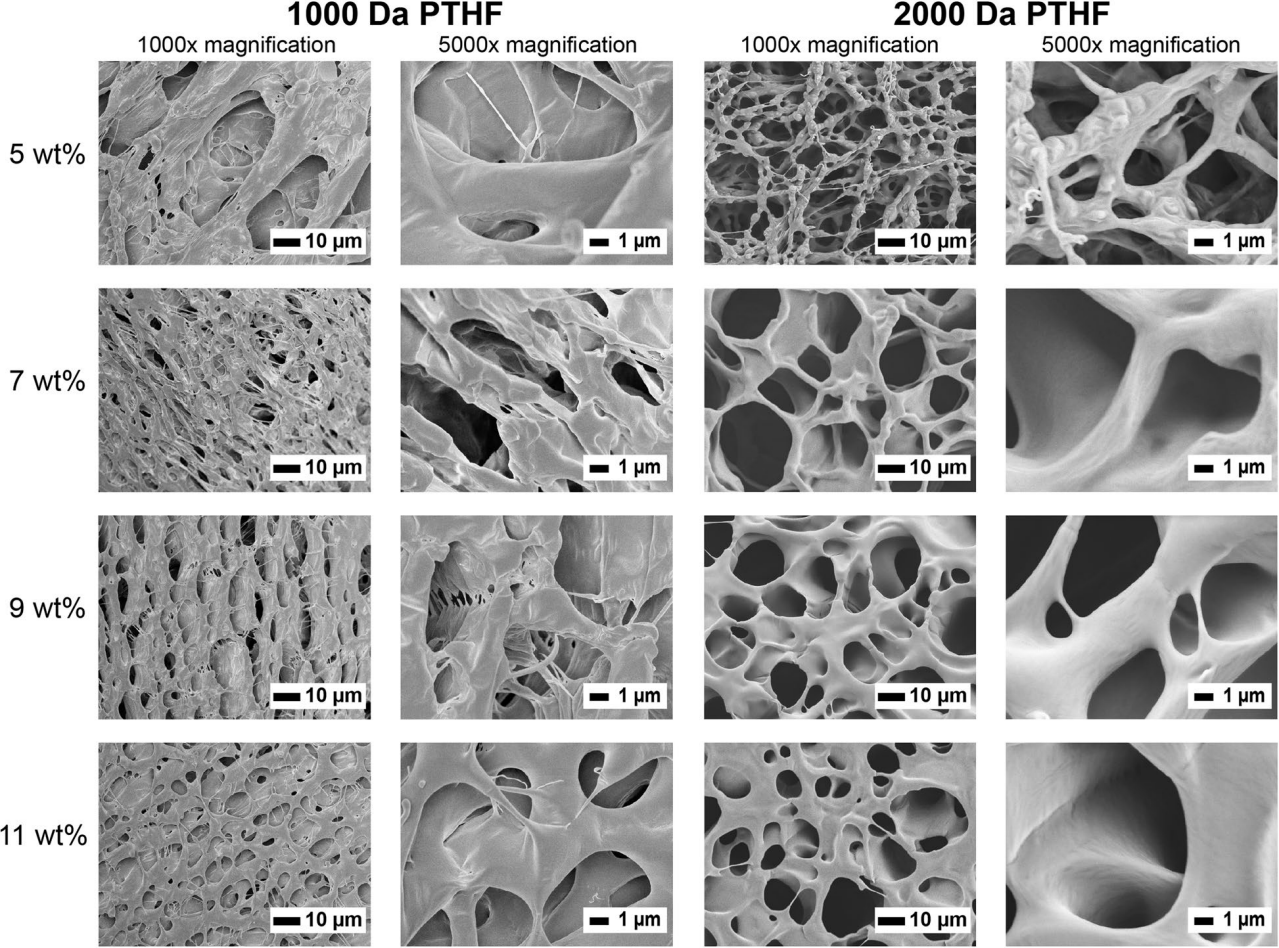
SEM characterization of the microstructure of EPT5 gels composed of either 1 kDa or 2 kDa PTHF at increasing copolymer concentration

To investigate the physically crosslinked network of EPT5 thermogels with either 1 kDa or 2 kDa PTHF incorporated, their microstructures were captured by using scanning electron microscopy (SEM) at different copolymer concentrations (Fig. 4). EPT5 with 1 T and 2 T showed significant difference of crosslinked network at the same copolymer concentration. Judging from the microscopic morphology, the crosslinked network structure of both two thermogels became tighter and stabler with increasing copolymer concentration and PTHF block length. This leads to an increase in the storage modulus and complex viscosity on a macroscopic scale.

**Fig. 5:**
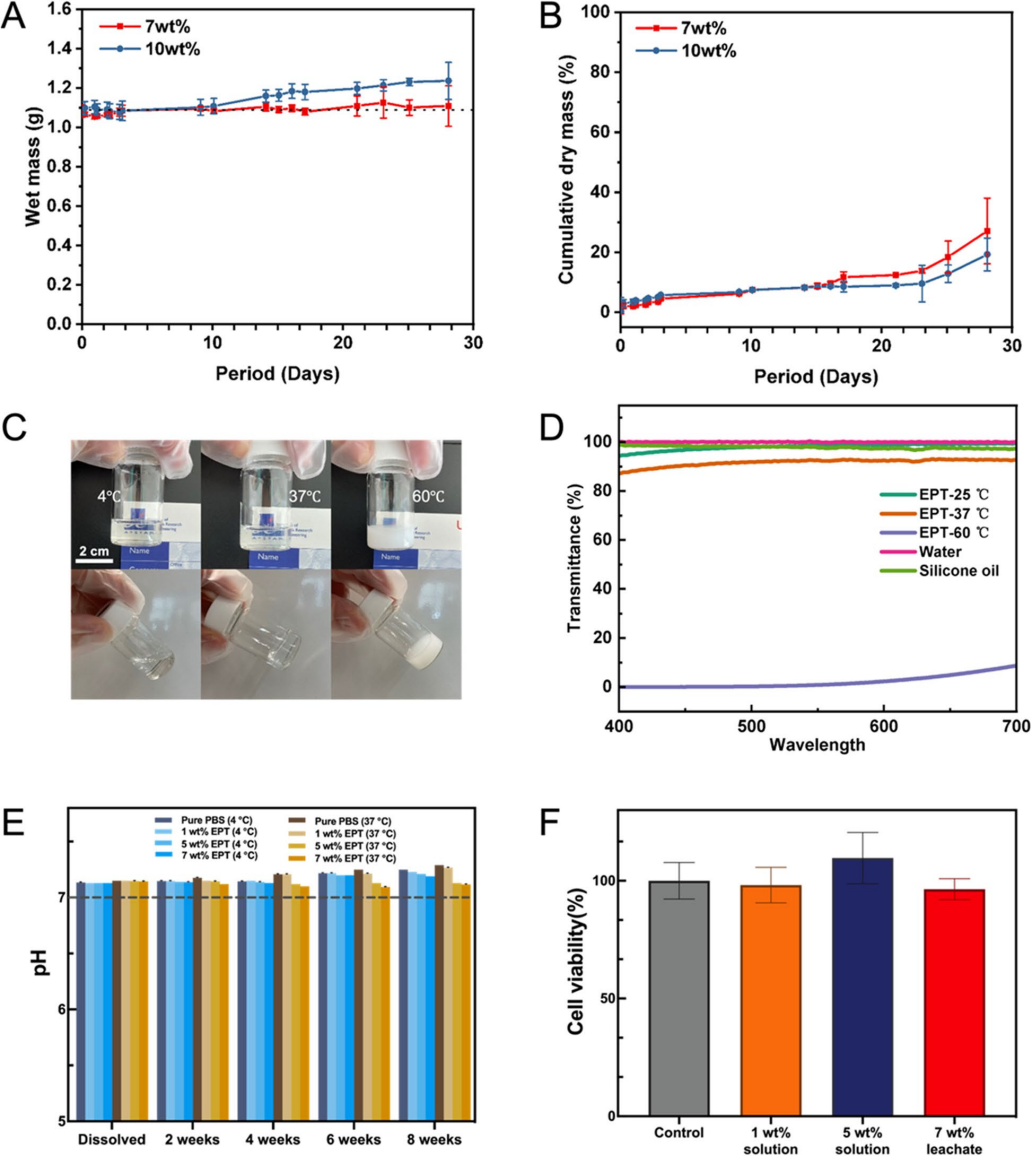
In vitro performances of EPT5-1 T thermogel. Gel erosion and drug release properties of EPT5-1 T thermogels: **A** Wet mass of EPT5-1 T thermogels at 7 wt% and 10 wt% (*n* = 3). **B** Cumulative dry mass loss from the EPT5-1 T thermogels at 7 wt% and 10 wt% (*n* = 3). Optical transparency and in vitro cell viability: **C** Pictures of 7 wt% EPT5-1 T at 4 ℃, 37 ℃, and 60 ℃; **D** UV–vis spectra of the transmittance of 7 wt% EPT5-1 T (at 25℃, 37 ℃ and 60 ℃), water, and silicone oil (both at 25 ℃). **E** pH stability of EPT5-1 T at 4 ℃ and 37 ℃ (*n* = 3). **F** Cell viability result of ARPE-19 cells incubated with EPT5-1 T thermogels (*n* = 6)

To investigate EPT5-1 T as a potential drug delivery depot, a gel erosion study was conducted. There was little change in wet mass for the 7 wt% gel, while the wet mass for the 10 wt% gel did not change for the first 10 days but started to show a small increase thereafter (Fig. 5A). We can see that an increase in rate of dry mass loss started occurring for both concentrations after 23 days of minimal mass loss, with only around 20% of dry mass lost after 28 days (Fig. 5B), which is indicative of bulk eroding polymers [51, 52]. As a combined result of hydrogel erosion and swelling, the 7 wt% gel showed a consistent wet mass with no obvious change in gel size, which could be an important property for administering into smaller spaces in vivo. To investigate application as a drug delivery depot, EPT-1 T was formulated at 10 wt% and in vitro release was conducted with the hydrophilic protein Bovine Serum Albumin (BSA). The release of BSA was shown to be relatively consistent and only close to 50% was released after 28 days (Figure S4). Therefore, EPT showed slow gel erosion and sustained release properties.

In the tube inversion experiments, we found that the optical transparency of EPT thermogel changes with temperature. To ensure that the 7 wt% EPT5-1 T thermogel can maintain high transparency at the body temperature, it was tested for transparency at different temperatures via visual observation and UV–vis transmittance check (Fig. 5C and 5D). The EPT5-1 T behaved as a clear solution and hydrogel at 4 ℃ and 37 ℃ respectively. Visual inspection demonstrated that the shape of the object (A*STAR Logo) can be clearly identified through the thermogel. In UV–vis spectra, its transmittance value remained around 90% at physiological temperature in the range of visible light, while the completely turbid gel was observed at 60 ℃. The refractive index is another important consideration for use in the eye. It was found to be around 1.3416 at room temperature and around 1.3446 at body temperature (Supplementary Table S1), which is close to the refractive index of the native human vitreous (~ 1.34) [5, 53].

To evaluate the potential for byproducts which could change the pH and lead to inflammatory reactions in the eye, EPT5-1 T solutions at concentrations of 1 wt% (low copolymer content), 5 wt% (high content but lower than CGC) and 7 wt% were chosen. We monitored the pH change over time and observed that their pH values were consistent with pure PBS after 8 weeks in vitro (Fig. 5E). As the results show in Fig. 5F, the cell viability remains above 95% for 24 h incubation. The copolymer exhibits very low toxicity in vitro, suggesting that the copolymer micelles do not show significant cytotoxicity to ARPE-19 cells at various concentrations and can be used in vivo.

### *In vivo* compatibility of 7 wt% EPT5-1 T thermogels in rabbits’ vitreous cavity

**Fig. 6:**
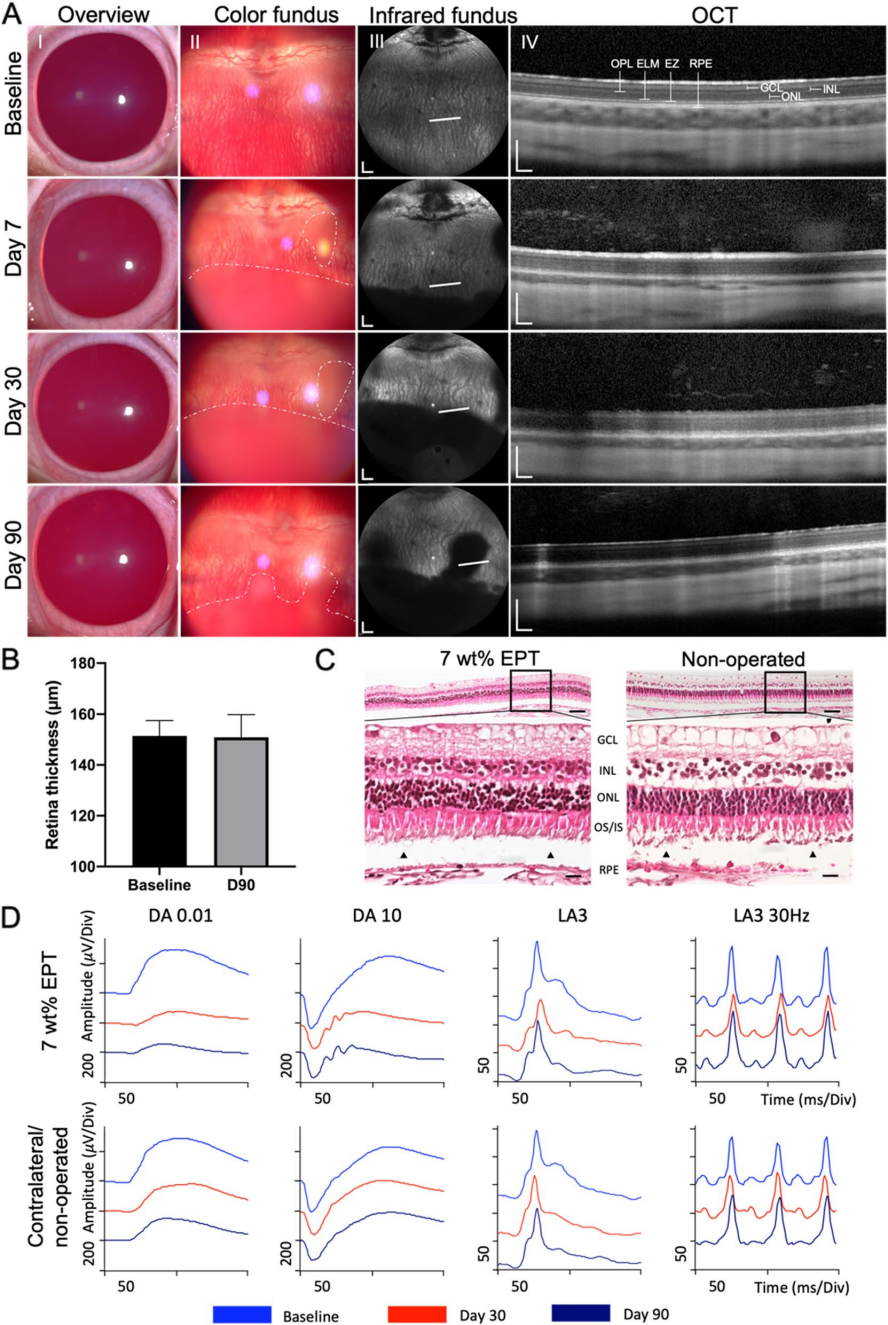
In vivo multimodal ophthalmic follow up of rabbit eyes injected with 7 wt% EPT5-1 T gels up to 3 months. **A** Ophthalmic multi-imaging assessments of one representative case of 7 wt% EPT gel. Column I, slit-lamp overview assessment showed negligible inflammation in the anterior chamber. Column II, color fundus images showed deposition of injected EPT gel at the inferior of the vitreous cavity (white dot lines). EPT gel was slightly opaque and blocked the view of fundus vessels. Column III, Infrared fundus images (820 nm wavelength) indicated that semi-opaque gels cast a black shadow on the fundus whilst floating in the vitreous cavity. White lines indicated the position where the OCT scans were taken. Column IV, cross section of retinal layers on OCT demonstrated normal retinal structures with the gel in-situ. **B** Quantification of full retinal thickness. Data represented as the mean ± SD, statistical analysis was performed using paired *t* test, *P* = 0.5072. **C** Histology (H&E) images confirmed normal retinal structures in 7 wt% EPT filled eyes compared to non-operated control. Upper panel was the overview of the retinal sample, while lower panel was the representative region in higher magnification. Black triangles indicate processing artifacts during the histology sample preparation. **D** Full field electroretinography (ERG) indicates preservation of visual function in a rabbit eye filled with 7 wt% EPT, with the contralateral eye as non-operated control. Scale bars,1 mm in infrared fundus, 200 µm in OCT, 50 µm in H&E upper panel, 10 µm in H&E lower panel. GCL = ganglion cell layer, INL = inner nuclear layer, ONL = outer nuclear layer, OPL = outer plexiform layer, ELM = external limiting membrane, EZ = ellipsoid zone, OS/IS = outer segment/ inner segment, RPE = retinal pigment epithelium

To evaluate the in vivo biocompatibility, the 7 wt% EPT5-1 T thermogels were injected into the vitreous cavity of rabbits to assess for biocompatibility. 25-gauge (G) core vitrectomy was performed on 9 eyes of 6 rabbits. Six eyes were filled with 7 wt% EPT5-1 T thermogel and 3 eyes with 20 wt% Pluronic F127, with 3 non-operated eyes as controls. All EPT or Pluronic thermogels were pre-cooled on ice and injected into the vitreous cavity using 25G needles without surgical complications. All samples formed gel in-situ within the vitreous cavity at body temperature. The biosafety of injected hydrogels was monitored using live, non-invasive, multimodal ophthalmic imaging. Both EPT and Pluronic thermogels showed negligible intraocular inflammation, with no cataract formation by slit lamp assessments (Fig. 6A and Supplementary Figure S5A) and intraocular pressure was maintained within the normal range of 9.2 to 15 mmHg). (Supplementary figure S6). The optically clear EPT thermogel was observed to turn slightly opaque at the point-of-injection and maintained opaque in the inferior vitreous cavity for 3 months (Fig. 6A). In contrast, 20% Pluronic F127 maintained optical clarity in the vitreous cavity post-surgery (Supplementary figure S5A).

Optical Coherence Tomography (OCT) images provides an optical tomographic technique based in vivo depth image of the retina. The hypo- and hyper- reflective bands on OCT could be correlated with the lamellar architecture and cellular constituents of the histology [54, 55]. Therefore, the maintenance of retinal thickness measurements on OCT images could be an indicator of retinal safety [44]. Although the long wavelength (820 nm) light was blocked by the EPT gels in the inferior vitreous cavity, the scans at the adjacent gel-media region demonstrated normal structure of the retina layers with maintenance of full retinal thickness (150.9 ± 9.0 μm at D90) compared to baseline (151.4 ± 6.0 μm, Fig. 6 A and B). Histology confirmed that normal retinal structure maintained both at the posterior pole and peripheral retina (Fig. 6C). In contrast, thinning of the retina with lost of visual function was observed in one of the three eyes filled with 20% Pluronic F127, although the gel remained transparent in vitreous cavity (Supplementary Figure S5). In addition, epiretinal membrane was observed during the follow up, which is a fibrocellular tissue formed on the inner surface of the retina, indicating an inflammatory response resulting in the proliferation of glial cells and fibrous astrocytes.

The decrease in retinal thickness is usually accompanied with loss in retina function which is undesirable [54]. A crucial criteria for successful vitreous implants for future clinical use is to ensure that the retinas maintain functional, and this can be assessed using electroretinography (ERG) functional assessment, which was performed on all rabbits monthly. The amplitudes and waveforms in the scotopic dark-adapted (DA) and photopic light-adapted (LA) spectrums indicated functional rod and cone cells respectively with preservation of waveform amplitudes corresponding to retained retina function [47]. Amongst all cases with thermogel injection, EPT cases (all 6 eyes) showed decreased ERG amplitude in scotopic (DA) but maintenance of amplitudes in photopic (LA) compared to baseline and non-operated eyes (Fig. 6D). Reduction in scotopic amplitudes might partially be affected by the opacity of EPT gel. This is in contrast to the 20 wt% Pluronic gel filled eyes, whereby one in three cases demonstrated complete loss of both scotopic and photopic waveform amplitudes, which also correlated with the OCT findings of thinning of all retinal layers in OCT images (Case #1 in Supplementary figure S5). In the other two cases, there was mild loss in both scotopic (DA) and photopic (LA) ERG spectrums, indicating a global toxic effect on both rods and cones in the retina.

### Analysis of gels harvested from vitreous

**Fig. 7:**
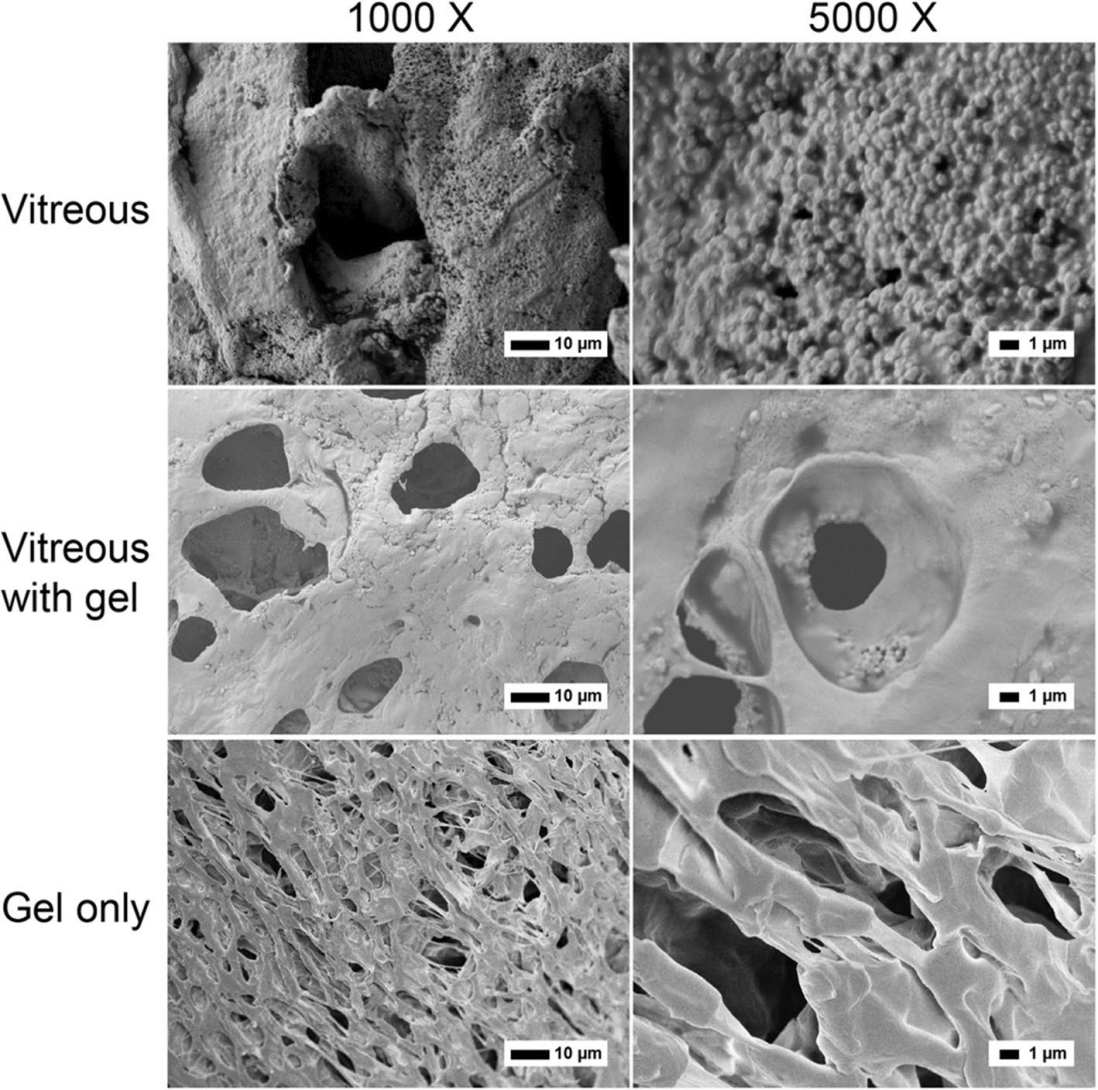
SEM analysis of gel samples after 3 months. Scale bar is 10 μm. The samples are non-operated vitreous, vitreous with gels injected after 3 months, and gel only samples

The vitreous was harvested from rabbits after 3 months for further analysis. Vitreous was flash frozen and the morphology was studied using SEM. The vitreous alone has round globular structures on its surface (Fig. 7). Gad-Elkareem et al. [56] observed a similar amorphous mass of globular proteins in the vitreous in the presence of microplasmin. In the vitreous with gel setup, the mesh-like network of the gel can be clearly seen, showing that the gel is still largely present within the vitreous even after 3 months. However, the gel after harvesting from vitreous looks distinctly different from gel alone, with thicker walls and larger rounded pores. There are also traces of rounded globular structures on the surface. Thicker walls have been previously observed in the presence of physiological concentration of salts such as sodium chloride, [57] so it is plausible that the changes seen is due to the interaction of the thermogel with vitreous fluids. Larger pores are also observed in EPT5-1 T gel at a lower concentration (Fig. 4), so gel erosion within the eye cavity could also contribute to the observed microstructure. SEM has similarly been used to analyse the morphology of chitosan hydrogels and hydrogel-coated PCL scaffolds before and after implantation and study the effects of in vivo interactions on the hydrogel [58, 59]. Overall, the SEM images suggest that the gel is mostly still present within the vitreous, but with changes in microstructure observed. Significantly, this is one of the first instances where the effect of in vivo implantation on changes in the microstructure of supramolecular hydrogels are studied.

**Fig. 8:**
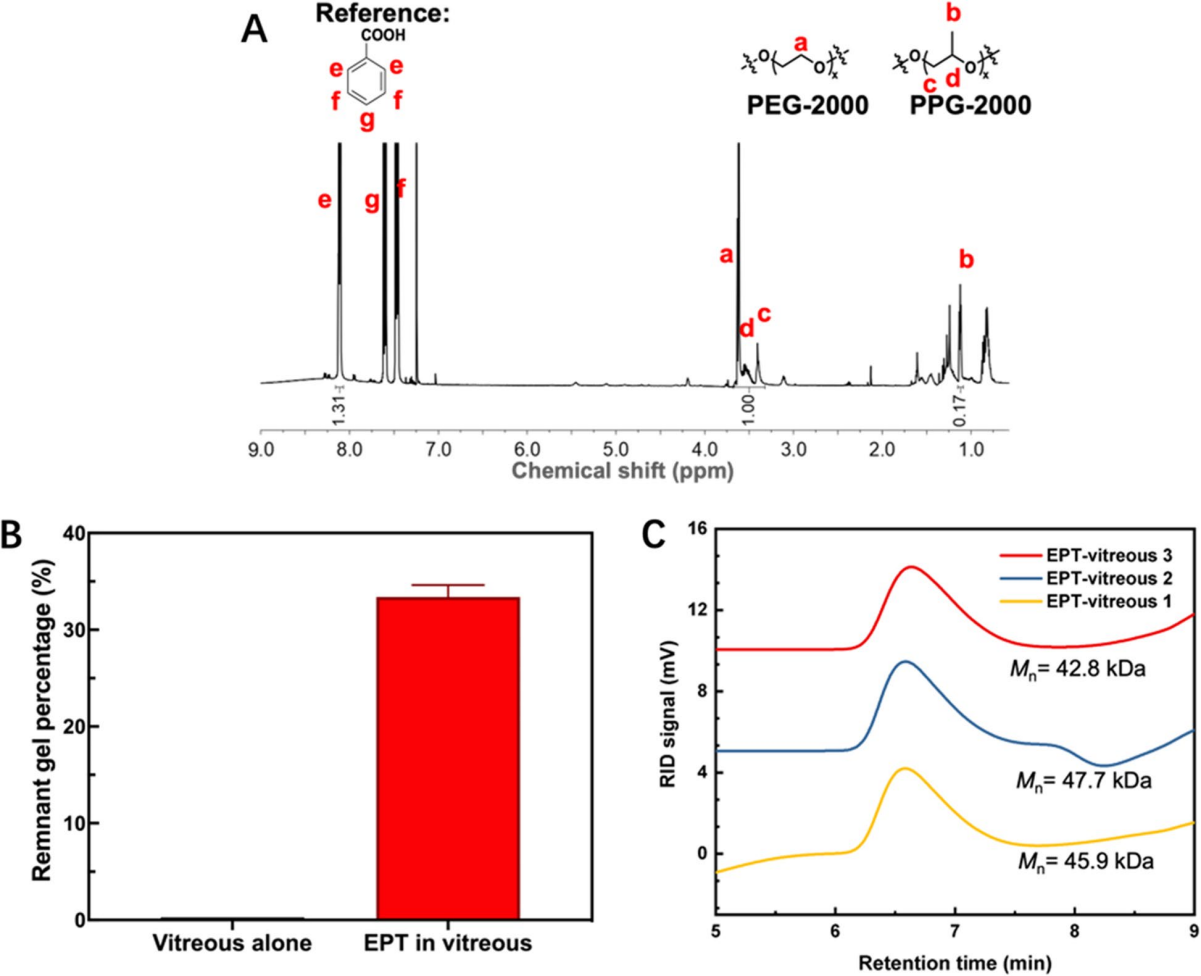
Polymer analysis in vitreous harvested after 3 months. **A** Representative quantitative ^1^H NMR of harvested vitreous in CDCl_3_ with 5 mg mL^−1^ benzoic acid as reference (protons are labelled accordingly on spectrum) was performed to quantify the amount of 7 wt% EPT5-1 T thermogel copolymer remnant in the vitreous. **B** Quantification of NMR results with benzoic acid as reference. (*n* = 3) **C** Retention time spectrum of the harvested vitreous (*n* = 3) was analysed using GPC with THF solvent. The harvested vitreous was lyophilised and extracted with chloroform first to isolate the polymer

The harvested gel samples were further characterized to understand polymer-level changes that had occurred. EPT5-1 T polymer was found to be still present in the vitreous after 3 months by the characteristic resonance peaks of PEG and PPG in water suppression ^1^H NMR (Supplementary Figure S7). To quantify the amount of polymer remaining, quantitative ^1^H NMR was performed with a known concentration of benzoic acid as reference (Fig. 8A, B, Supplementary Figure S8). It was found that there was 33.4 ± 1.2% of EPT polymer remaining in the vitreous. This is substantially higher than the more degradable EPCG polymer, with around 30% remaining after 1 month, but with all the polymer cleared out after 4 months [60]. To determine if the polymer integrity was maintained, the polymer components from the vitreous was extracted with chloroform. GPC analysis showed that the molecular weight did not change substantially after recovery from the vitreous over 3 months (Fig. 8C, Supplementary Figure S9, Supplementary Table S2). This suggests that there was only a small amount of polymer degradation that occurred, while the mechanism for polymer loss from the vitreous was likely predominantly through gel erosion.

## Discussion

In this study, we found that it is possible to tune the PTHF thermogels by varying both the weight percent and block molecular weight of PTHF segment in copolymer, and that this affects the gelation temperature, storage modulus and the injectability of the gel itself. The EPT5-1 T gel showed slow gel erosion and sustained release properties, and minimal cell viability change when it was applied to retinal cell culture in vitro. We chose 7 wt% EPT thermogel for in vivo experiment. Because thermogels with higher copolymer concentration possess higher swelling ratios, there is higher swelling counter-force against the eye cavity and this could lead to the elevation of IOP [44]. Compared to the in vitro swelling result of 10 wt% thermogel, the change in wet mass of the 7 wt% EPT thermogel is small, which we believe is achieved due to its swelling-erosion balance, so that it can preclude any potential problems with intraocular pressure after injection. The 7 wt% EPT thermogel showed desirable transparency properties at room temperature in vitro. But it turned translucent in color fundus imaging and blocked the longwave length light (Infrared fundus imaging) after injection. This suggested the light in visible wavelengths could still reach the retina which is correlated with retinal function tests. The ERG waves amplitudes only slightly decreased during scotopic recordings and maintained in photopic. In addition, the histology findings also confirmed normal retinal structure in the region exposed to EPT gels, which further confirmed the intraocular safety to use EPT thermogel. From our previous study, we have demonstrated that opaque hydrogels, especially those crosslinked by hydrophobic association, can be optimised further to get transparency close to native vitreous [61]. Thus, there is room for EPT hydrogel to be further improved its transparency towards clinical use as a long-term vitreous tamponade. There was also negligible inflammation, chemical induced cataract formation, raised intraocular pressure or any other sign of major side effects. This contrasted with 20 wt% Pluronic F127 gels, which was consistent with our previous reports, whereby biocompatibility issues such as loss of retinal structures was observed [44]. Taken together, these biosafety tests suggested that the EPT thermogels can be a potentially viable option as an injectable hydrogel for intraocular application.

The incorporation of the bioinert block PTHF in our EPT gels provided key benefits. Hydrolysis of polyester blocks in biodegradable gels could produce byproducts which change the pH, possibly leading to a mild to severe inflammation [62]. In our case, a significant advantage is that EPT thermogels are bioinert with no additional hydrolysable blocks incorporated which could affect pH. The in vivo clearance mechanism is not through breakage of the polymer backbone chains, as the polymers were shown to be largely intact with no significant change in the polymer molecular weight before and after implantation in the vitreous. Therefore, there is less risk of instability or other chemical changes in vivo. Using SEM, the gel network microstructure was shown to be maintained, although it was different from the gel microstructure pre-implantation. Despite their bio-inertness, EPT can still be naturally removed from the eye by bio-erosion, as seen when ~ 70% of the original EPT was cleared from the vitreous after 3 months. This is consistent with the results of the in vitro gel erosion assay, where only around 25% of the EPT polymer was removed from the erosion setup after a month. The cleared EPT gel would also likely be replaced with regenerated vitreous, as shown from our previous study [44]. To sum up, EPT can be mostly naturally cleared and has the advantage of likely not requiring surgical intervention for removal, unlike silicon oil endotamponades which are the clinical standard of care.

There is a clinical need for a sustained release drug delivery system for posterior disease, and long-term vitreous substitutes (over 3 months) providing tamponade effects for complicated retinal detachment patients [63]. The clinical standard of care for AMD and PDR is monthly intravitreal injection of anti-VEGFs such as Aflibercept, so drug delivery systems that release on the order of months are of clinical interest [64]. The EPT gel was shown to be stable and demonstrated sustained release properties when formulated at 10 weight percent, with a relatively consistent release profile and close to 50% released only after 1 month. This is significantly slower than previous injectable hydrogels which showed close to 50% release of larger antibody proteins after just 10 days, and the release duration can likely be extended further by increasing the polymer concentration [65]. Current gaseous tamponades (e.g. SF6, C2F6, C3F8) have a short residence time and only works for simple retinal detachments, while silicone oil has a longer residence time (3 to 6 months), but has to be removed with a second surgery to prevent silicone oil complications [66]. Therefore, it is worthwhile to develop a vitreous tamponade with a longer biodegradation time for more complicated cases of retinal detachment. The EPT thermogel system is designed with a biostable block (PTHF) in the polymer chains, and shows a higher residence stability in the eye. NMR results showed that around 30% of EPT polymers were still present in vitreous cavity at 3 month follow up, while Pluronic F127 or EPC gels were completely degraded and non-detectable at 3 months post-operation [44]. Theoretically, this biostable version of thermogels may achieve the upper limit of residence stability in the eye for our physically crosslinked hydrogels. It will expand the potential intraocular application need of the thermogel system in addition to our previous thermogel system for mid-term (1–3 months) use [44]. Ophthalmic imaging allowed us to non-invasively track the status of the EPT thermogel over 90 days. In addition to EPT having higher stability, this study allows us to better visualize and understand the long-term behavior of supramolecular biomaterials after implantation into the eye, and the gel structural changes that occur due to interactions between the materials and in situ microenvironment in the rabbit vitreous cavity. Due to the limitation of the current experiment design, we chose 3 months as the follow-up end point, which was consistent with our previous reports of the EPC gel system. There is much that is relatively unknown that can be further investigated, such as the longest duration that EPT thermogels can stay within the vitreous in vivo and the pathway for gel clearance from the eye.

## Conclusion

In summary, a series of multiblock EPT thermosensitive hydrogels were synthesized, and after selection using multiple characterization tools, we found that the 7 wt% EPT5-1 T copolymer has desirable properties for intraocular application. Its high-water content, injectability, suitable storage modulus, slow gel erosion, refractive index, and in vitro transparency close to native vitreous are all consistent with its use as a promising intraocular implant. We carried out comparative studies of EPT with the commercial Pluronic F127 thermogel and found that the EPT gel exhibits better biosafety and long-term biocompatibility. While the EPT gel is more stable in vivo and can provide a longer support compared to PCL-based thermogels, it can still be mostly naturally cleared from the vitreous. We believe that with further optimization, it will be a competitive candidate for clinical use as a long-term intraocular implant.

## Supplementary Information

**Additional file 1: Supplementary Figure 1.** 500 MHz 1H NMR spectroscopy of (A) EPT5-1T and (B) EPT5-2T. **Supplementary Figure S2.** Raw absorbance spectrum and fit diagram of CMC calculation (A) EPT3-1T; (B) EPT7-1T; (C) EPT3-2T; (D) EPT5-2T. **Supplementary Figure S3.** TEM image of the EPT5-1T thermogel micelle. **Supplementary Figure S4.** In vitro release of bovine serum albumin from 10 wt% EPT5-1T thermogels. **Supplementary Table 1**. Refractive index analysis. **Supplementary Figure S5.** *In vivo* multimodal ophthalmic follow up of rabbit eyes injected with 20 wt% Pluronic gels. (A) Ophthalmic multi-imaging assessments of the eye with 20 wt% Pluronic gel, case #1 (flat ERGs in B). Column I, slit-lamp overview assessment showed negligible inflammation in the anterior chamber. Column II, color fundus images showed transparent Pluronic gel in the vitreous cavity with clear retinal vessels presented. Column III, the clear infrared fundus images showed clear fundus structure. White lines indicated the position where the OCT scans were taken. Column IV, cross section of retinal layers on OCT showed thinning retinal layers from INL to RPE layers. White arrows indicated epiretinal membrane formed above the retina. (B) Full field electroretinography (ERG) assessments of 3 cases of 20 wt% Pluronic gel injected eyes. Case #1 showed complete flat ERG waves, while the other two cases showed decreased waves in all four channels compare to baseline. Scale bars,1 mm in infrared fundus, 200 µm in OCT. GCL= ganglion cell layer, INL= inner nuclear layer, ONL = outer nuclear layer, OPL = outer plexiform layer, ELM = external limiting membrane, EZ = ellipsoid zone, RPE = retinal pigment epithelium. **Supplementary Figure S6.** Intraocular pressure follow-up post operation. The intraocular pressure was maintained within the normal range (9.2 to 15 mmHg). **Supplementary Figure S7.** Representative water suppression ^1^H NMR spectrum of harvested vitreous with EPT hydrogel at 3 month. The characteristic peaks due to PEG and PPG (indicated above) were observed suggesting the presence of remnant EPT copolymer in the vitreous cavity. **Supplementary Figure S8.** Quantitative ^1^H NMR of harvested vitreous of non-operated control at 3 month in CDCl_3_ with 5 mg ml^-1^ benzoic acid as reference (protons are labelled accordingly on spectrum). This was used as a control to quantify the NMR signals for EPT thermogel copolymers in the non-operated vitreous cavities. **Supplementary Figure S9.** GPC retention times of original EPT copolymers. **Supplemental Table S2.** Molecular weight of 7 wt% EPT5-1T copolymers harvested from vitreous after 3 months.

## Data Availability

The datasets used and/or analyzed during the current study are available from the corresponding author on reasonable request.
