# Supplementary material for: Injectable PTHF-based thermogelling polyurethane implants for long-term intraocular application
Source: Biomater Res. 2022 Dec 2;26:70. doi: 10.1186/s40824-022-00316-z (PMC9716749; doi:10.1186/s40824-022-00316-z)
Supplement: Supplementary file 1 — Additional file 1: Supplementary Figure 1. 500 MHz 1H NMR spectroscopy of (A) EPT5-1T and (B) EPT5-2T. Supplementary Figure S2. Raw absorbance spectrum and fit diagram of CMC calculation (A) EPT3-1T; (B) EPT7-1T; (C) EPT3-2T; (D) EPT5-2T. Supplementary Figure S3. TEM image of the EPT5-1T thermogel micelle. Supplementary Figure S4. In vitro release of bovine serum albumin from 10 wt% EPT5-1T thermogels. Supplementary Table 1. Refractive index analysis. Supplementary Figure S5. In vivo multimodal ophthalmic follow up of rabbit eyes injected with 20 wt% Pluronic gels. (A) Ophthalmic multi-imaging assessments of the eye with 20 wt% Pluronic gel, case #1 (flat ERGs in B). Column I, slit-lamp overview assessment showed negligible inflammation in the anterior chamber. Column II, color fundus images showed transparent Pluronic gel in the vitreous cavity with clear retinal vessels presented. Column III, the clear infrared fundus images showed clear fundus structure. White lines indicated the position where the OCT scans were taken. Column IV, cross section of retinal layers on OCT showed thinning retinal layers from INL to RPE layers. White arrows indicated epiretinal membrane formed above the retina. (B) Full field electroretinography (ERG) assessments of 3 cases of 20 wt% Pluronic gel injected eyes. Case #1 showed complete flat ERG waves, while the other two cases showed decreased waves in all four channels compare to baseline. Scale bars,1 mm in infrared fundus, 200 µm in OCT. GCL= ganglion cell layer, INL= inner nuclear layer, ONL = outer nuclear layer, OPL = outer plexiform layer, ELM = external limiting membrane, EZ = ellipsoid zone, RPE = retinal pigment epithelium. Supplementary Figure S6. Intraocular pressure follow-up post operation. The intraocular pressure was maintained within the normal range (9.2 to 15 mmHg). Supplementary Figure S7. Representative water suppression 1H NMR spectrum of harvested vitreous with EPT hydrogel at 3 month. The characteristic peaks du [file 40824_2022_316_MOESM1_ESM.docx]

Supplementary information

**Injectable PTHF-based thermogelling polyurethane implants for long-term intraocular application**

Kaiwen Zhang^1, 2, #^, Zengping Liu^3,4,5, #^, Qianyu Lin^1^, Yi Jian Boo^1^, Valerie Ow^1^, Xinxin Zhao^4^, Daniel Soo Lin Wong^3^, Jason Y.C. Lim^1^, Kun Xue^1,^*, Xinyi Su^3,4,5,6,^*, Decheng Wu^2,^*, Xian Jun Loh^1,7,^*


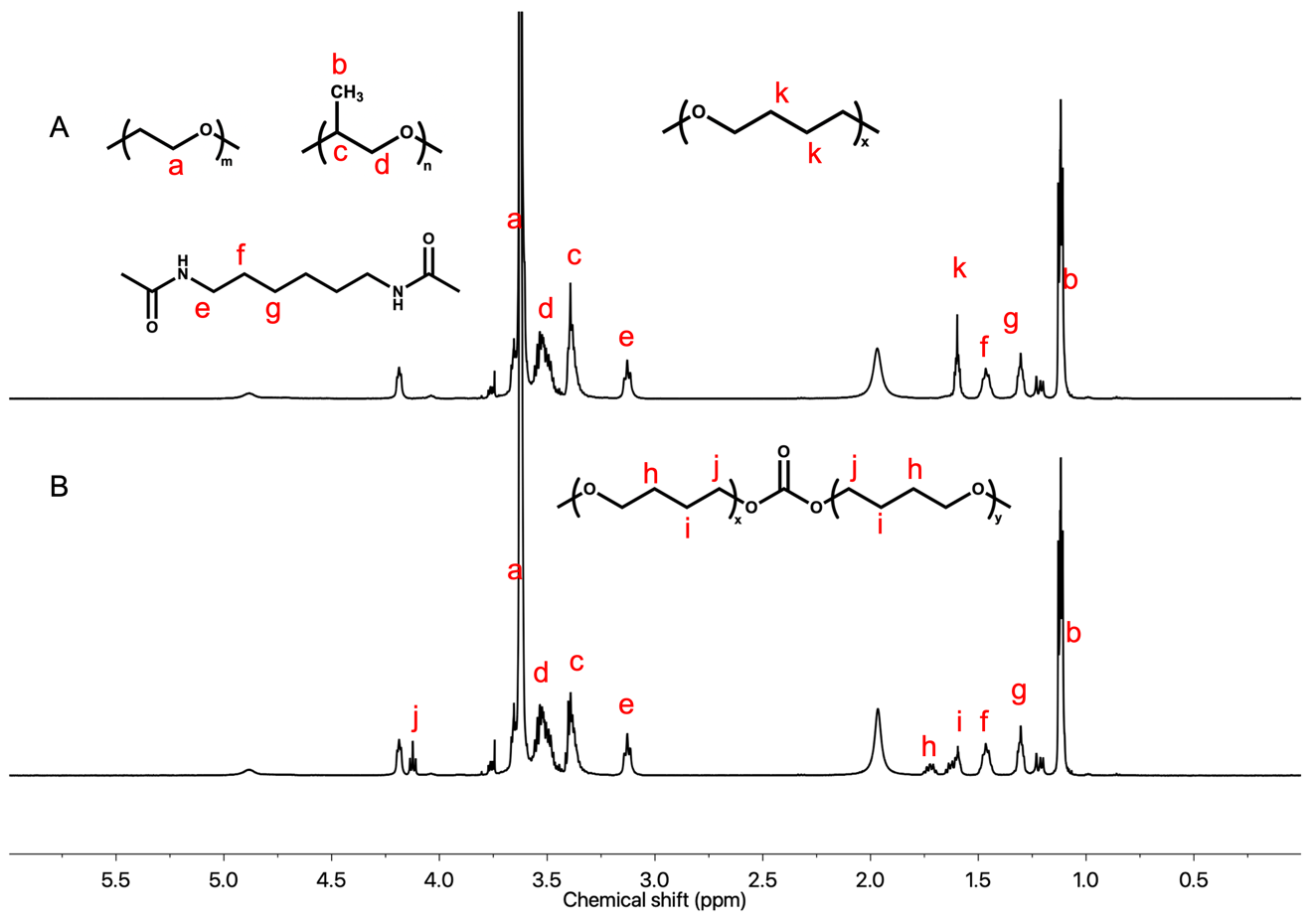


**Supplementary Figure 1.** 500 MHz 1H NMR spectroscopy of (A) EPT5-1T and (B) EPT5-2T.


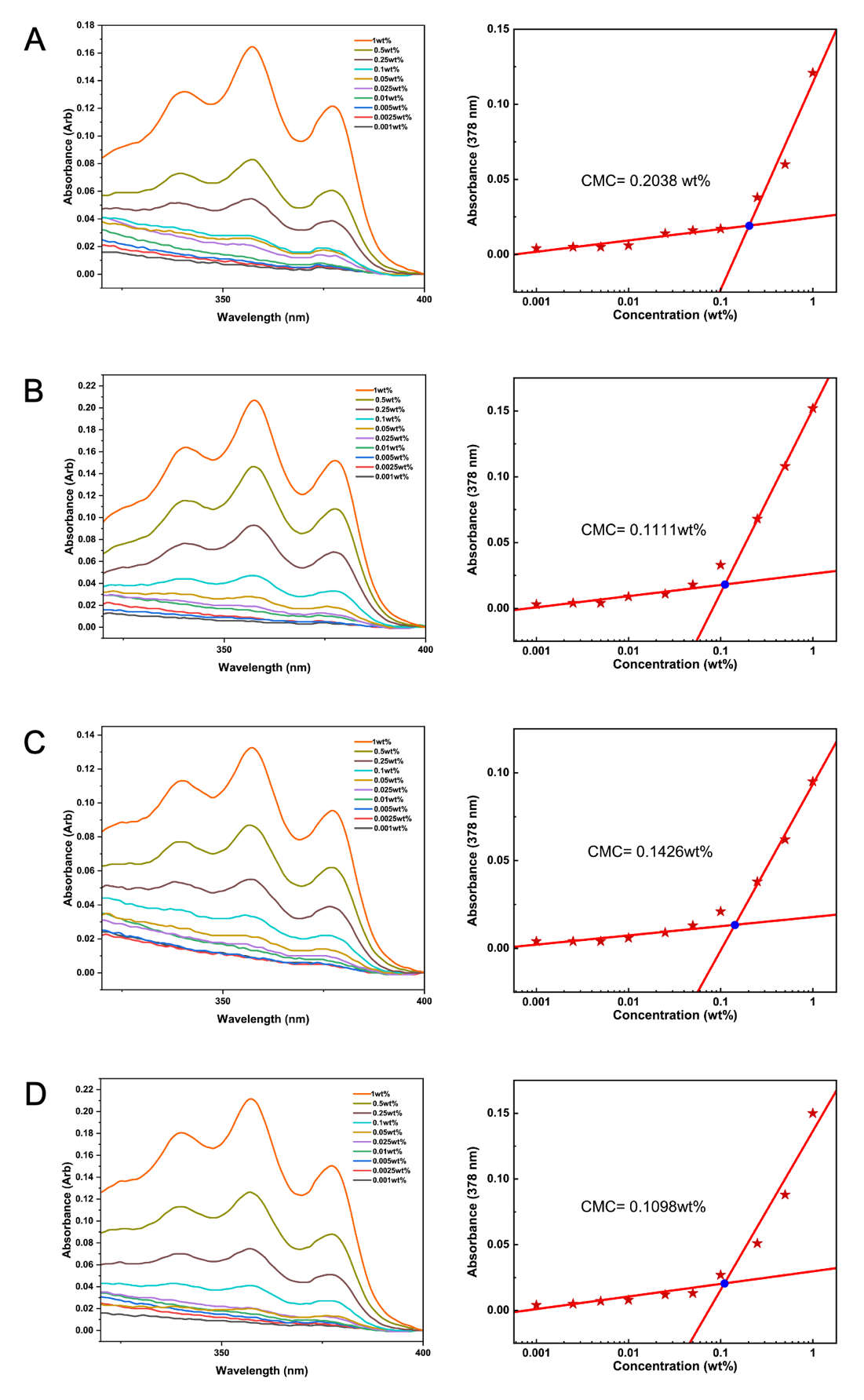


**Supplementary Figure S2.** Raw absorbance spectrum and fit diagram of CMC calculation (A) EPT3-1T; (B) EPT7-1T; (C) EPT3-2T; (D) EPT5-2T.

**
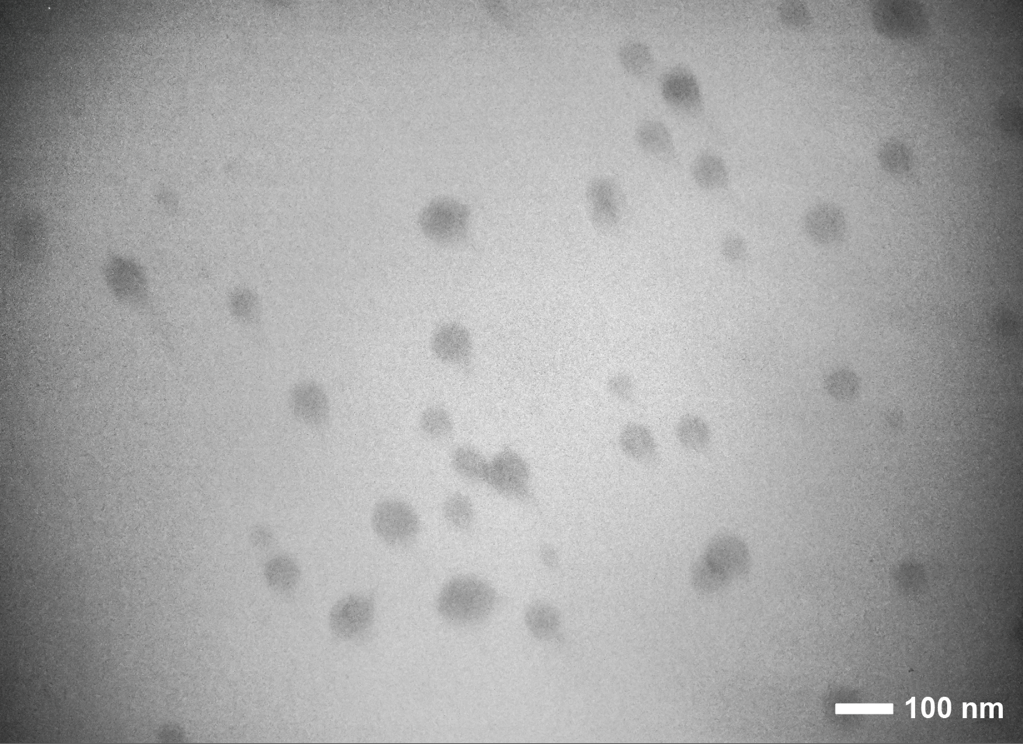
**

**Supplementary Figure S3.** TEM image of the EPT5-1T thermogel micelle.


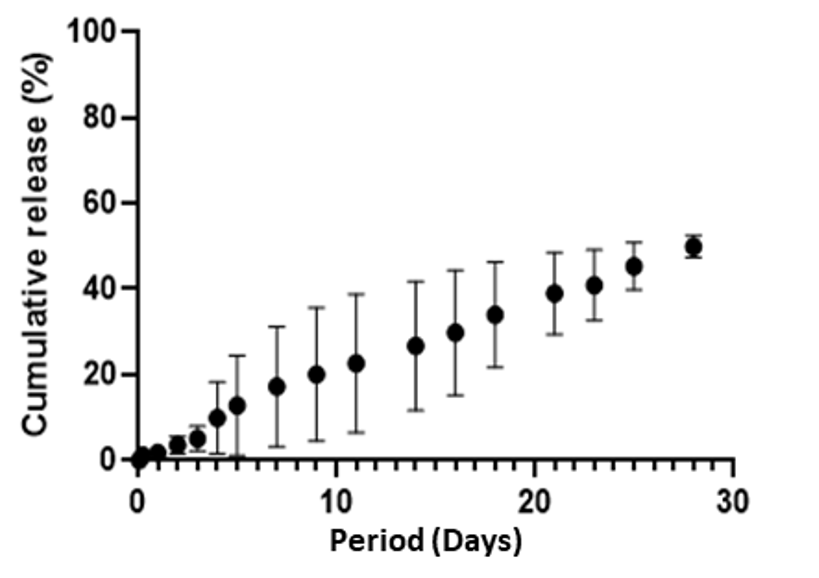


**Supplementary Figure S4.** In vitro release of bovine serum albumin from 10 wt% EPT5-1T thermogels.

**Supplementary Table 1**. Refractive index analysis

| Temperature/°C | Refractive index ^a^ | |
| --- | --- | --- |
| 25 | 1.3416 |  |
| 37 | 1.3446 |  |

[a] Determined with an Abbe refractometer with a temperature control system (n=3)


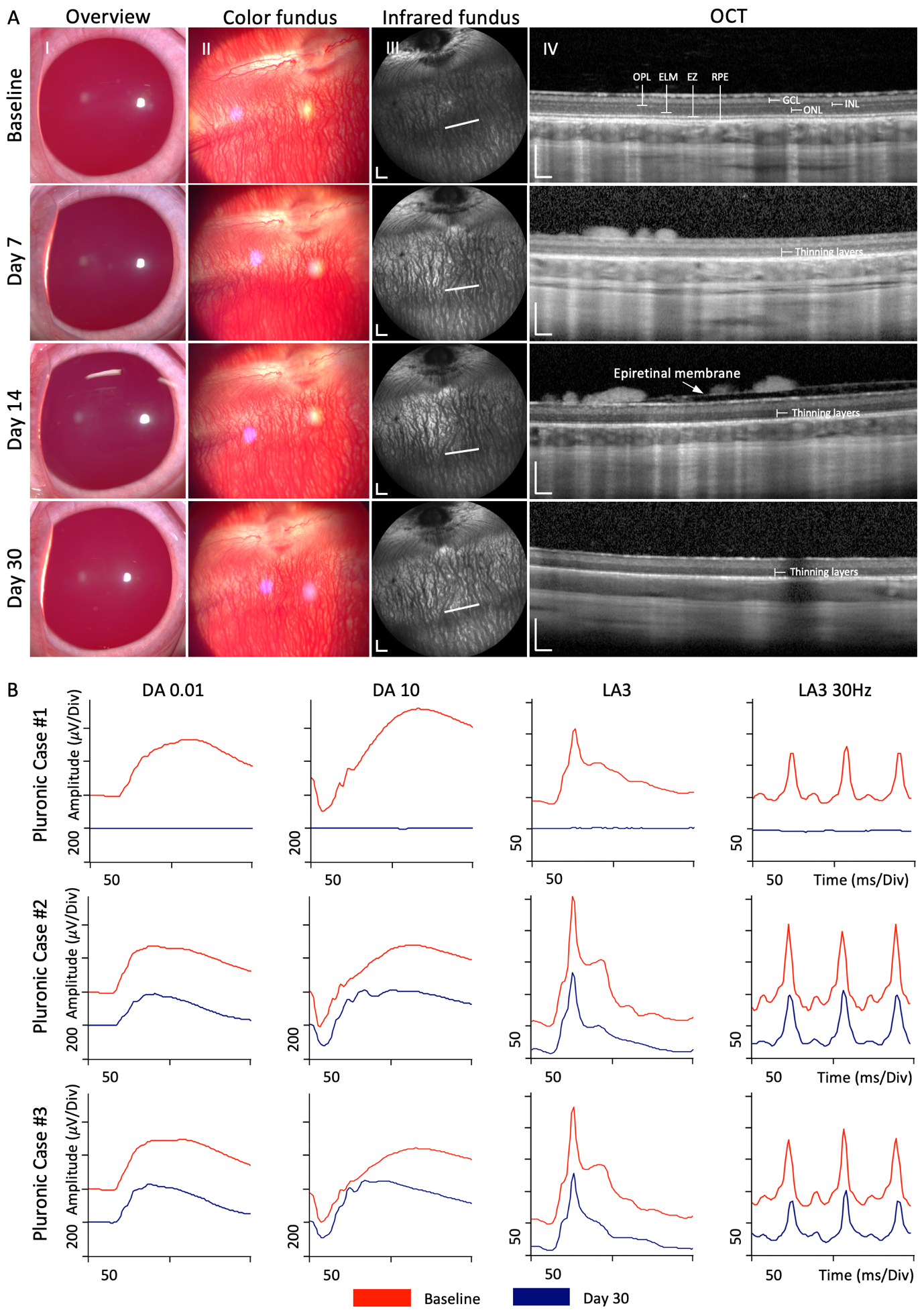


**Supplementary Figure S5.** *In vivo* multimodal ophthalmic follow up of rabbit eyes injected with 20 wt% Pluronic gels. (A) Ophthalmic multi-imaging assessments of the eye with 20 wt% Pluronic gel, case #1 (flat ERGs in B). Column I, slit-lamp overview assessment showed negligible inflammation in the anterior chamber. Column II, color fundus images showed transparent Pluronic gel in the vitreous cavity with clear retinal vessels presented. Column III, the clear infrared fundus images showed clear fundus structure. White lines indicated the position where the OCT scans were taken. Column IV, cross section of retinal layers on OCT showed thinning retinal layers from INL to RPE layers. White arrows indicated epiretinal membrane formed above the retina. (B) Full field electroretinography (ERG) assessments of 3 cases of 20 wt% Pluronic gel injected eyes. Case #1 showed complete flat ERG waves, while the other two cases showed decreased waves in all four channels compare to baseline. Scale bars,1 mm in infrared fundus, 200 µm in OCT. GCL= ganglion cell layer, INL= inner nuclear layer, ONL = outer nuclear layer, OPL = outer plexiform layer, ELM = external limiting membrane, EZ = ellipsoid zone, RPE = retinal pigment epithelium.


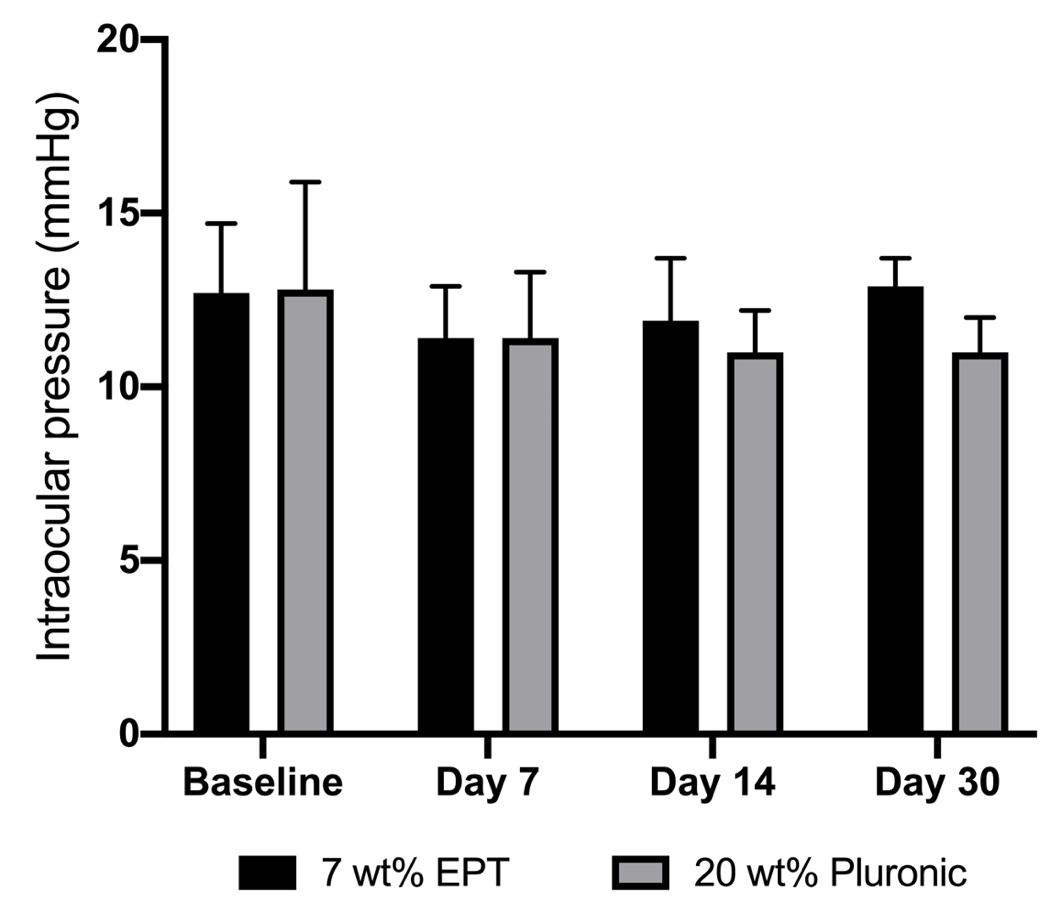


**Supplementary Figure S6**. Intraocular pressure follow-up post operation. The intraocular pressure was maintained within the normal range (9.2 to 15 mmHg)


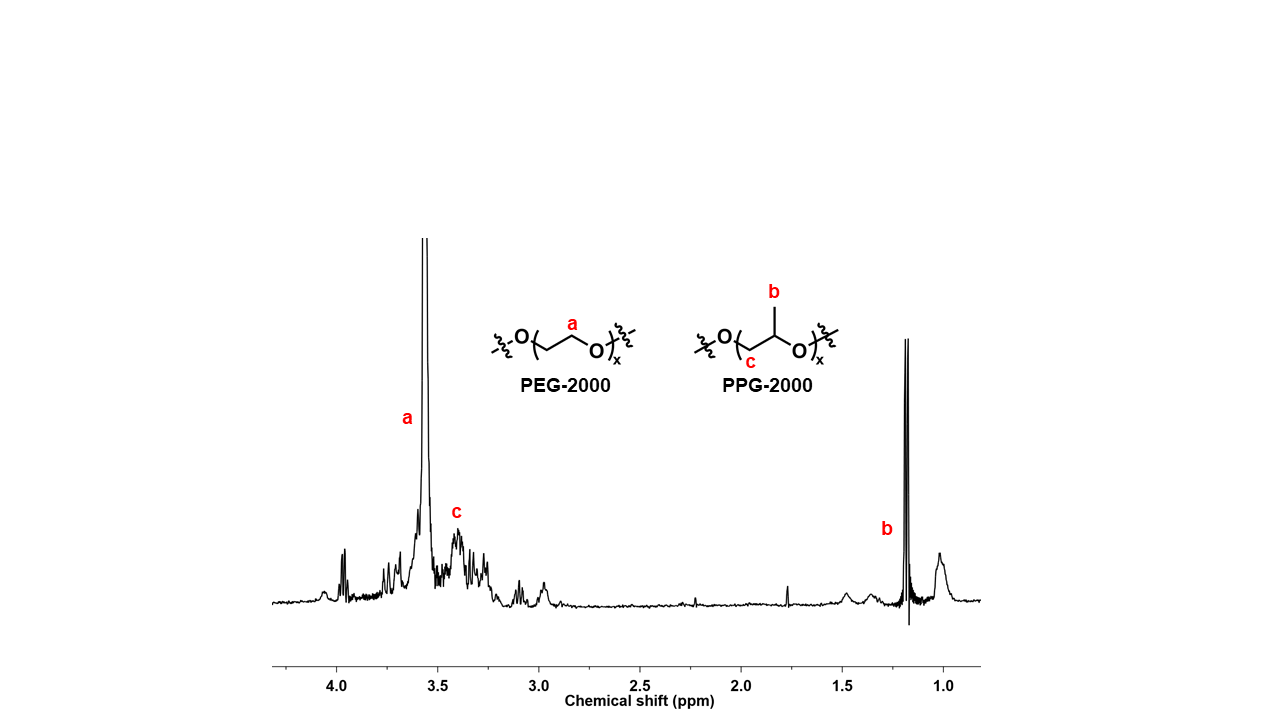


**Supplementary Figure S7**. Representative water suppression ^1^H NMR spectrum of harvested vitreous with EPT hydrogel at 3 month. The characteristic peaks due to PEG and PPG (indicated above) were observed suggesting the presence of remnant EPT copolymer in the vitreous cavity.


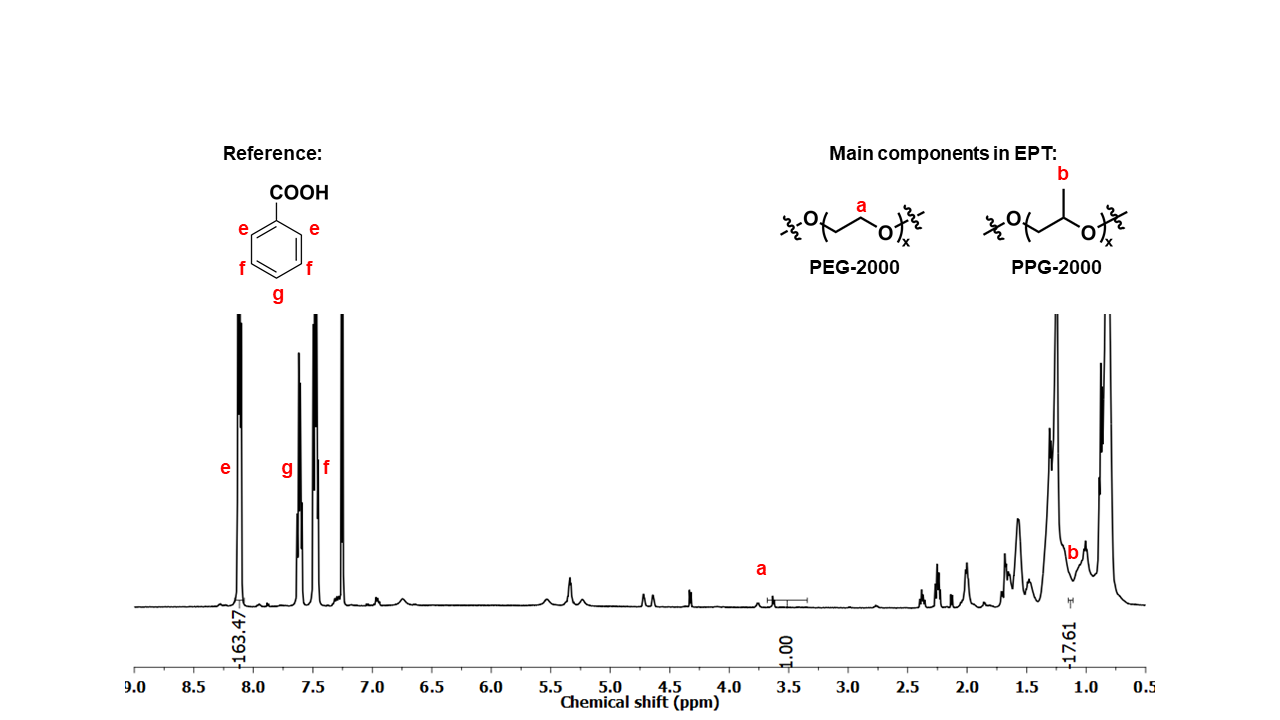


**Supplementary Figure S8**. Quantitative ^1^H NMR of harvested vitreous of non-operated control at 3 month in CDCl_3_ with 5 mg ml^-1^ benzoic acid as reference (protons are labelled accordingly on spectrum). This was used as a control to quantify the NMR signals for EPT thermogel copolymers in the non-operated vitreous cavities.


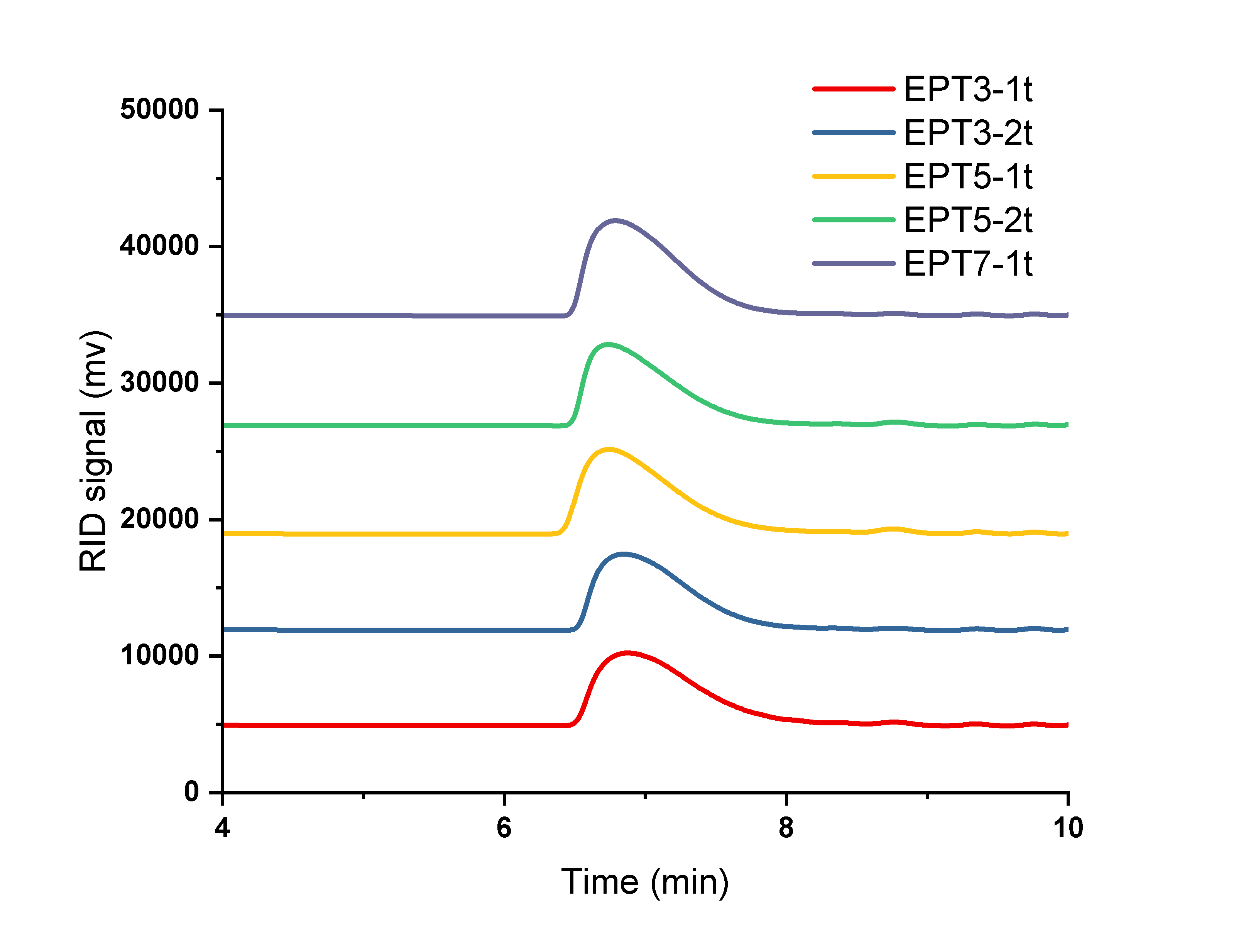


**Supplementary Figure S9.** GPC retention times of original EPT copolymers.

**Supplemental Table S2**. Molecular weight of 7 wt% EPT5-1T copolymers harvested from vitreous after 3 months.

|  |  | **Molecular weight^a^** | | |
| --- | --- | --- | --- | --- |
| Sample |  | M_n_ (kDa) | M_w_ (kDa) | PDI |
| EPT-vitreous 1 |  | 45.9 | 58.0 | 1.26 |
| EPT- vitreous 2 |  | 47.7 | 58.4 | 1.23 |
| EPT- vitreous 3 |  | 42.8 | 53.8 | 1.26 |

[a] vitreous was extracted with chloroform, dried and dissolved in THF before performing gel permeation chromatography with THF as solvent and polystyrene standards.
